# Serviceability Moment Capacity of Bolted Endplate Minor-Axis Connections in Prefabricated Steel Frames: Role of Column Web Yielding and Numerical Verification

**DOI:** 10.3390/ma19153165

**Published:** 2026-07-23

**Authors:** Abudureyimujiang Aosimanjiang, Mo Chen, Zhiyu Wang, Qunyi Huang

**Affiliations:** 1 College of Civil Engineering, Kashi University, Kashi 844006, China; 2 Xinjiang Key Laboratory of Engineering Materials and Structural Safety, Kashi 844006, China; 3 CREGC Architectural & Construction Engineering Co., Ltd., Chengdu 610083, China; 4 School of Architecture and Environment, Sichuan University, Chengdu 610065, China; 5 College of Civil Engineering, Southwest Jiaotong University, Chengdu 610031, China

**Keywords:** minor-axis connection, yield line theory, reliability analysis, plastic mechanism

## Abstract

This paper aims to systematically investigate the out-of-plane yield performance, structural reliability, and sensitivity boundaries of minor-axis joints under construction overloads. To achieve this objective, a high-fidelity three-dimensional non-linear finite element framework incorporates precise solid continuum elements, contact non-linearities, and multi-linear material models, which is successfully validated against referenced experimental curves. Utilizing efficient Latin Hypercube Sampling integrated with response surface surrogate methodologies, a comprehensive stochastic parametric scanning is conducted to map the multi-dimensional scatter profiles and probabilistic capacity responses at targeted elastic thresholds. Furthermore, based on the upper-bound theorem of plasticity, closed-form analytical formulations defining the competition between independent and global plastic mechanisms are established and rigorously validated against extensive numerical parametric matrices. The core mechanical insights demonstrate that the column web thickness *t*_w_ and column section depth *b* overwhelmingly dictate nearly 60% of the joint’s elastic-limit resistance by directly defining the out-of-plane bending span. While the divergence between the two competitive plastic mechanisms remains minute under severe flange constraints, the research uncovers a critical localized interaction: in connections with a relatively thick *t*_w_, the extended endplate thickness *t*_ep_ and endplate flexibility trigger a dynamic migration of the rotation axis between the longitudinal boundaries *L*_0_ and *L*_a_, significantly shortening the effective internal lever arm. For practical design applications, calibrating these formulations with a partial safety factor *γ*_M_ = 1.25 successfully establishes a dependable lower-bound design strength, providing a verified safety red-line for temporary cantilevered scaffolding installations.

## 1. Introduction

**Figure 1:**
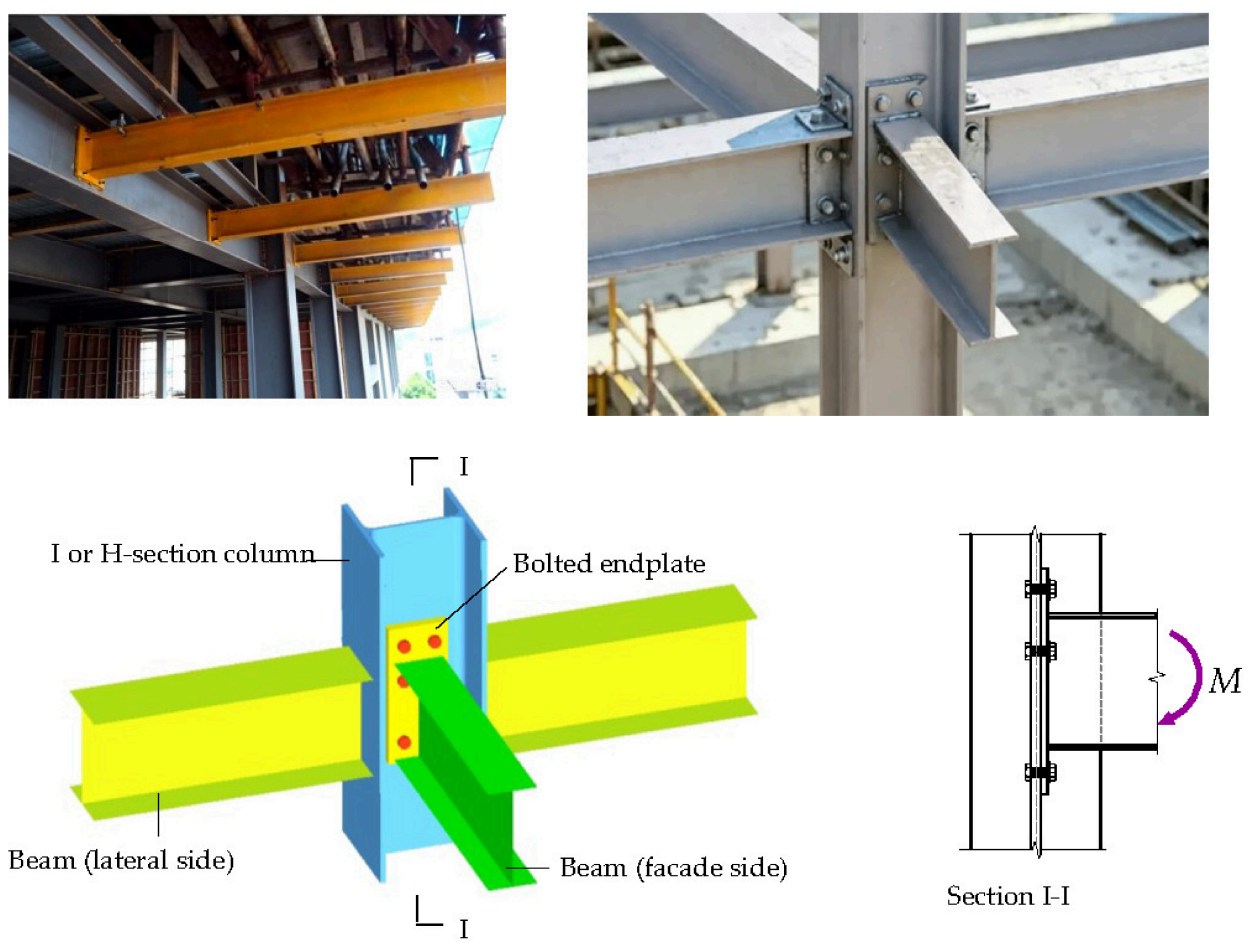
Typical bolted endplate minor-axis connections under moment loading.

Bolted endplate connections to H-section column webs, commonly referred to as minor-axis or weak-axis connections, are critical structural components widely employed in modern multi-high-rise steel buildings, industrial workshops, and temporary construction facilities. To capture their realistic implementation, Figure 1 introduces typical joint topologies natively photographed by the authors during generic structural field investigations. As mapped out in these configurations, when a cantilevered beam transfers bending moments (*M*) directly to the minor axis of an H-section column, the flexible column web is subjected to severe out-of-plane bending, resulting in a distinct semi-rigid behaviour. To track the structural macro-evolution across these advanced beam-column spatial configurations, recent landmark investigations highlight critical technological milestones alongside remaining mechanical gaps. Nie et al. [1] numerically evaluated U-shaped connector-enhanced endplate configurations attached to H-section weak-axis columns, demonstrating that the U-connector expands the panel zone boundary and materially upgrades shear resistance thresholds, leading to optimized structural criteria for connector/endplate thickness ratios. Mata and Nuñez [2] examined the multi-axial cyclic behaviour of concrete-filled tube (CFT) columns integrated with bidirectional moment connections under local slenderness effects, verifying that while high local slenderness parameters penalize the cyclic strength, stable structural ductility and robust energy dissipation can be successfully preserved below a 3% story drift ratio limit. Furthermore, Lu et al. [3] analysed the systemic influence of floor slab composite actions on the seismic performance of weak-axis connections, proving that the continuous rigid constraint offered by composite slabs limits the out-of-plane warping distortion of the steel components and stabilizes full-field plastic dissipation pathways. Collectively, these pioneer works underscore that while endplate mechanics for H-section weak-axis beams are increasingly developed through innovative hardware modification [1], bidirectional coupling [2], or composite interactions [3], minor-axis behavioural characterization under localized boundary variations remains far less developed than its strong-axis counterparts. Resolving this discrepancy is critical because properly accommodating these flexible weak-axis boundaries represents an invaluable asset to activate bidirectional frame spatial coupling and systemic structural redundancy, resolving a vital scientific gap for multi-hazard engineering resilience.

In recent years, the application of these connections has extended beyond permanent main frames to temporary structural systems, such as the widely adopted cantilevered basket-type scaffolding systems (flower-basket scaffolding) and temporary cantilevered platforms supporting construction floors. In these temporary installations, the cantilevered beams or primary frames are directly anchored to the existing structural column webs via minor-axis bolted endplates. Although the nominal design loads for these temporary construction installations are relatively small compared to primary structural frames, they are frequently exposed to severe operational uncertainties. Severe overloading events, induced by the accidental accumulation of construction debris, concentrated material stockpiling, or dynamic construction impacts, are highly likely to occur. Because the out-of-plane stiffness of the column web is inherently low, such accidental overloads can abruptly trigger localized yielding or premature plastic failure of the connection, posing a significant threat to the overall stability of the construction platform and risking catastrophic progressive collapse.

While this flower-basket scaffolding scenario serves as the primary engineering catalyst and a high-risk loading case for this investigation, the structural implications of this study extend far beyond into universal steel mechanics. The joint configuration, characterized by eccentrically delivered out-of-plane forces transferring through high-strength bolt rows directly into a flexible, spatially confined steel plate, presents a classical yet highly complex boundary-value problem. Therefore, utilizing this temporary structure scenario provides an ideal, highly conservative benchmark to test the upper bounds of yielding theory. The resulting closed-form mathematical equations and stochastic sensitivity laws quantified herein possess fundamental applicability, offering critical design insights for both temporary construction stabilization and permanent steel frame minor-axis connections subjected to accidental overloads.

Despite their widespread application, contemporary structural design codes fail to provide comprehensive or optimized guidelines for minor-axis endplate connections, particularly those utilizing multi-row or double-row bolt configurations. For instance, design specifications such as Eurocode 3 (EN 1993-1-8) [4], AISC 360 [5], and the Chinese Standard for Design of Steel Structures (GB 50017-2017) [6] offer highly mature, standardized procedures based on the “component method” for major-axis joints (where beams connect to column flanges), as systematically presented in Table 1. However, for minor-axis connections, these codes either simplify the behaviour as purely pinned joints or offer overly conservative equations that fail to capture the complex out-of-plane plate-to-web interaction. This regulatory lacuna stems from the highly non-linear deformation field of the column web bounded by two rigid column flanges, which creates an extreme sensitivity to geometric and material variations.

To bridge this gap, extensive numerical and experimental research has been conducted over the past decade to characterize the behavioural nuances of semi-rigid minor-axis or weak-axis joints. Oh et al. [7] evaluated the seismic behaviour of weak-axis column-tree moment connections with reduced beam sections, demonstrating that while such configurations exhibit ductile response up to 5% story drift ratio and effectively mitigate brittle failures near stub welds, their global efficiency is heavily sensitive to complex field welding quality. Nuñez et al. [8] numerically investigated the bidirectional cyclic response of weak-axis end-plate moment connections under biaxial loading, demonstrating that while beams connected to the minor axis show partially restrained behaviour, the joint rotation remains closer to a pinned classification than a fully rigid baseline. This flexible boundary significantly impacts the global drift limits and multi-story frame stability under lateral cyclic actions. Yılmaz [9] proposed and validated via finite element analysis a bolted end-plate connection with a T-adapter for weak-axis beam-to-column joints, concluding that although stiffer T-adapter models achieve rigid joint classification, significant plastic rotation capacity, and energy dissipation potential, such a configuration introduces substantial geometric complexity and added execution costs. Lu et al. [10] introduced the box-shaped panel zone end-plate connection designed for weak-axis columns, and they conducted an analysis of the seismic performance of joints equipped with stiffeners at the beam ends. The findings indicated that incorporating a stiffener within the joint significantly enhances both the stiffness and load-bearing capacity of the connection. However, these noteworthy prior investigations are inherently limited by their shared analytical boundaries. On the one hand, they primarily evaluate the global hysteretic performance under cyclic seismic indicators, structural drift optimization, or specific specialized deterministic configurations. Consequently, their macro-scale seismic findings fail to address a critical structural localized hazard—namely, the competitive independent vs. global out-of-plane punching yielding of the flexible column web when subjected to severe, localized quasi-static accidental construction overloads. On the other hand, these existing experimental or numerical investigations are predominantly deterministic, failing to account for the stochastic nature of geometric parameters encountered in real-world construction environments. Under these non-seismic overload scenarios, the structural demand transitions from seismic drift accumulation to the continuous spatial mapping of localized yield lines, where a standalone deterministic safety factor is insufficient to evaluate the true safety margin of minor-axis configurations. Consequently, reliability analysis and parametric sensitivity assessments are urgently required to quantify how these geometric variations impact the structural resistance. Recently, response surface methods (RSM) and sampling techniques (e.g., Latin Hypercube Sampling) have gained traction in structural engineering to explore multi-variable spaces without the prohibitive computational costs of direct Monte Carlo simulations [11]. In minor-axis configurations, the scatter plots generated via these parametric responses can reveal the non-linear coupling effects between variables—such as whether increasing the endplate thickness yields diminishing returns if the column web thickness remains constant. Quantifying these global sensitivity indices is crucial for establishing “red line” safety boundaries for temporary scaffolding attachments.

To precisely articulate the remaining scientific lacuna, a foundational distinction must be established across three progressive structural tiers: (i) Conventional strong-axis endplate connections are extensively regulated by design standards and existing literature, operating under the macro-mechanical assumption that beam-end plastification serves as the primary governing energy-dissipation mechanism. (ii) In sharp contrast, minor-axis or weak-axis connections transfer bending demands through the flexible, spatially confined column web. This shifts the failure paradigm from global beam plastification to a localized out-of-plane plate web-yielding mechanism, which demands a completely distinct mechanical interpretation and separate design justification due to the complex plate-to-flange interaction fields. (iii) Crucially, current code-based approaches fail to provide a comprehensive, continuous mapping of how multi-dimensional geometric variations modify these weak-axis failure typologies, leaving a severe research void regarding systematic parametric sensitivity and stochastic uncertainty evaluations.

While refined finite element (FE) parametric analysis provides macroscopic trend insights, it remains an inconvenient tool for rapid engineering checks or direct daily design. This underscores the necessity of analytical prediction methods, among which the Yield Line Theory (YLT) serves as the most powerful upper-bound plastic mechanism approach. Originally developed for concrete slabs, YLT was adapted for steel beam-to-column joints by pioneering researchers like Stockwell [12], Packer et al. [13] and Yardimci et al. [14]. For minor-axis connections, however, the yield lines are confined within the rectangular boundary of the column web, bounded by the stiff column flanges as integrated into mainstream design. Researchers like Gomes et al. [15] and Lima et al. [16] successfully formulated yield line mechanisms for single-row bolted minor-axis connections, providing accurate predictions for localized plastic failure. Recently, Ramzi et al. [17] developed a modified yield-line model for eccentric double longitudinal plate connections to square hollow section T-joints, accounting for the contribution of chord sidewall yielding and proposing improved design equations to address the conservatism of current codes that only consider chord face plastification. Nevertheless, when engineering demands mandate higher moment resistance, such as in heavy cantilevered scaffolding brackets, a double-row bolt configuration must be deployed. The introduction of a second row of bolts fundamentally complicates the plastic mechanism. In a double-row configuration, the connection can fail via two distinct conceptual routes: an independent plastic mechanism, where each bolt row develops its own isolated yield line loop without interfering with the adjacent row, or a global plastic mechanism, where the rows cooperate to form a single, encompassing yield line perimeter around the entire bolt group. While recent literature has attempted to classify these mechanisms in generic plates, a rigorous analytical derivation tailored to bolted H-section minor-axis web constraints remains completely absent. Understanding the competition between these two mechanisms is vital, although physical intuition suggests that due to the tight geometric constraints of the column web, the energetic paths of both mechanisms may yield close proximity. A precise quantification of their micro-differences is necessary to validate whether a unified, simplified design formula can be confidently recommended to field engineers.

To resolve these outstanding issues, this paper is built upon the scientific hypothesis that under accidental construction overloads, the flexible column web acts as the primary energy-dissipation component, and its extreme spatial confinement by rigid flanges forces a convergence between independent and global plastic mechanisms. To maintain this specific focus, the overall research boundary is defined by assuming that: (i) structural failure is strictly isolated within the minor-axis joint zone, (ii) peripheral boundary members, such as beams, possess sufficient capacity to remain within their elastic range during the localized overloading event, and (iii) the structural evaluation is governed by the critical static limit state, isolating the intrinsic quasi-static resistance of the connection to establish the absolute safety red-line prior to progressive collapse.

To systematically validate this hypothesis, the research is executed through three logical stages. First, an advanced non-linear finite element model is developed in ANSYS (Version 2019 R1) and meticulously validated against established experimental database curves to confirm its accuracy in capturing the *M*-*θ* relations and out-of-plane web yielding fields. Utilizing efficient Latin Hypercube Sampling, a comprehensive parametric study is conducted to generate multi-dimensional scatter plots and trend responses. Rather than focusing on pure mathematical fitting, these responses are used to elevate the physical understanding of plate-to-web stiffness ratios and identify the most sensitive design parameters under construction overloads. Second, based on the upper-bound theorem of plasticity, closed-form analytical equations are derived for both the independent and global plastic mechanisms of double-row bolted minor-axis configurations. The minute differences between these two theoretical mechanisms are critically evaluated and verified against the extensive ANSYS parametric database. Finally, based on the physical convergence of these mechanisms, streamlined and practical design recommendations are proposed to ensure the structural safety of both permanent frames and high-risk temporary construction scaffolding systems.

## 2. Numerical Modelling and Parameter Analysis

### 2.1. Finite Element Mesh and Boundary Configurations

**Figure 2:**
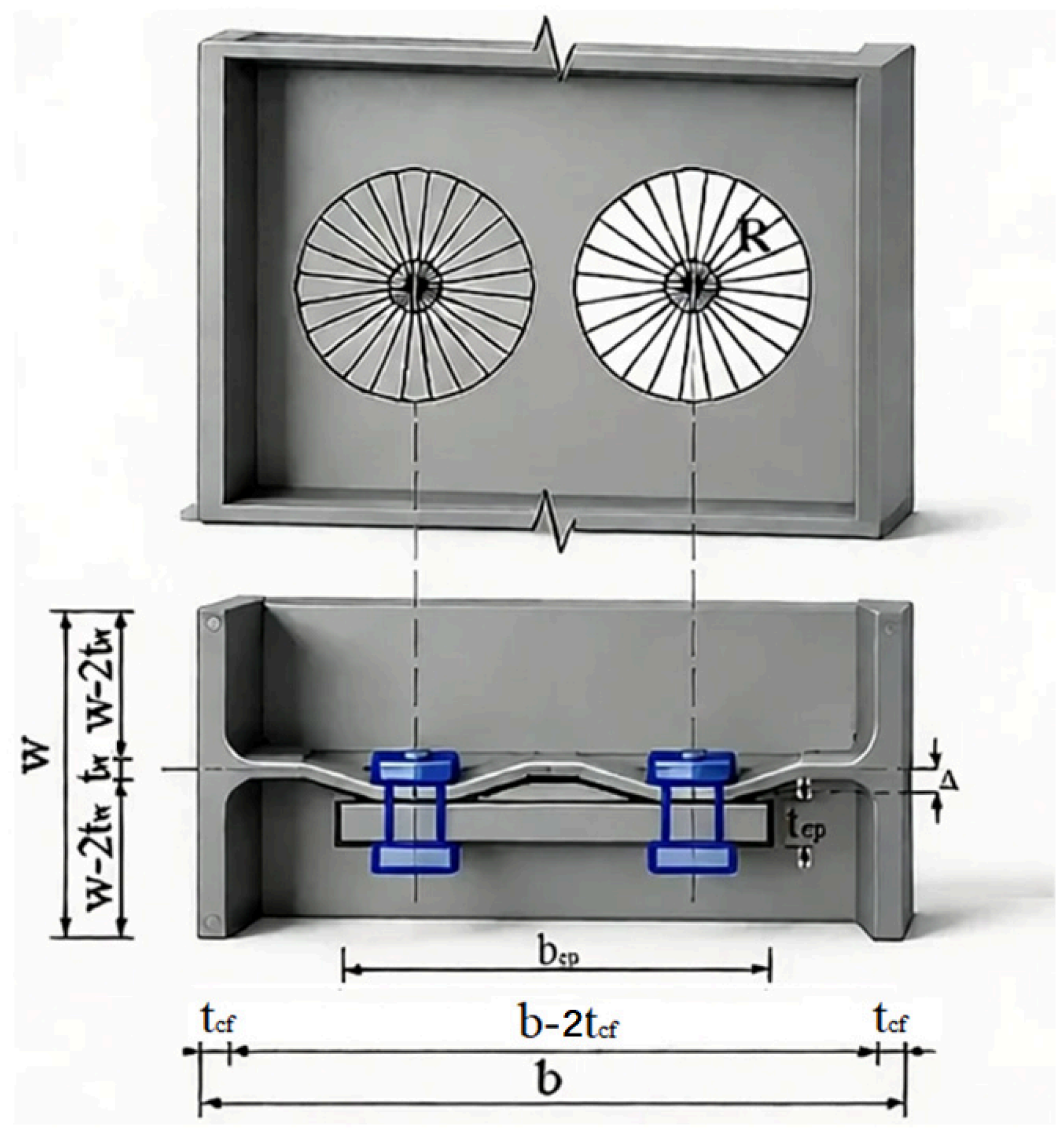
Illustration of geometric parameters of analytical connection with classical yield pattern (circular fan zones around bolts indicate localized plastic punching fields).

The geometric configuration and parametric definitions of the double-row bolted endplate connection to the H-section column web (minor-axis connection) are schematically illustrated in Figure 2. To facilitate independent interpretation of the kinematics, the arrows superposed on the web plates specify the admissible virtual displacement trajectories and the shifting boundaries of the plastic hinge lines. Crucially, the circular fan zones surrounding the bolt cluster explicitly represent the highly localized plastic punching shear fields that develop through the flexible column web plate during deep plastic states. To establish a rigorous analytical and numerical framework, the primary structural components are defined by a comprehensive set of geometric variables, which also serve as the core factors in the subsequent reliability and sensitivity assessments. The H-section column is characterized by its total section depth *b*, flange thickness *t*_cf_, and web thickness *t*_w_. The extended endplate is defined by its total width *b*_ep_ and thickness *t*_ep_. The high-strength bolt assembly is defined by the nominal bolt diameter *D*_b_ and the horizontal spacing designated as *β*_0_*b*_0_. To satisfy the dual objectives of this study, ensuring a physically verified simulation framework and assessing large-scale structural uncertainties under construction loads, two distinct sets of geometric dimensions are utilized: (1) Validation Specimen (Neves et al. [18]): To rigorously validate the baseline accuracy of the numerical simulation methodology, an explicit experimental minor-axis joint tested by Neves et al. [15] was replicated. This validation specimen utilizes a European standard configuration based on Eurocode 3 (EN 1993-1-8) [4] column cross-section of HEA 220, a beam cross-section of IPE 200, an extended endplate with dimensions of 276.8 × 117 × 20 mm, and high-strength bolts with a nominal diameter *D*_b_ = 16 mm spaced at a vertical/horizontal pitch *β*_0_*b*_0_ = 60 mm. The selection of this European standard configuration provides a well-documented benchmark with clear out-of-plane web yielding failure mechanisms. (2) Reliability Benchmark Specimen: To better reflect the realistic, heavy-duty load conditions encountered in modern engineering field practices—particularly for cantilevered basket-type scaffolding attachments (flower-basket scaffolding)—a larger-scale benchmark connection was established for the subsequent parametric reliability and yield line analysis. This model features a column height of 1200 mm with a robust cross-section of H400 × 400 × 10 × 16 (*b* = 400 mm, *t*_w_ = 10 mm, *t*_cf_ = 16 mm), a cantilevered beam length of 800 mm with a cross-section of H300 × 150 × 6 × 10 mm, an extended endplate of 460 × 180 × 20 mm, and high-strength bolts with *D*_b_ = 20 mm and a standardized spacing *β*_0_*b*_0_ = 100 mm. H400 × 400 × 10 × 16 is the hot-rolled H-section steel (Maanshan Iron & Steel Co., Ltd., Maanshan, China). Scaling up from the European test configuration to this heavy H-section geometry bridges the gap between laboratory validation and real-world construction site vulnerability under accidental overloads.

**Figure 3:**
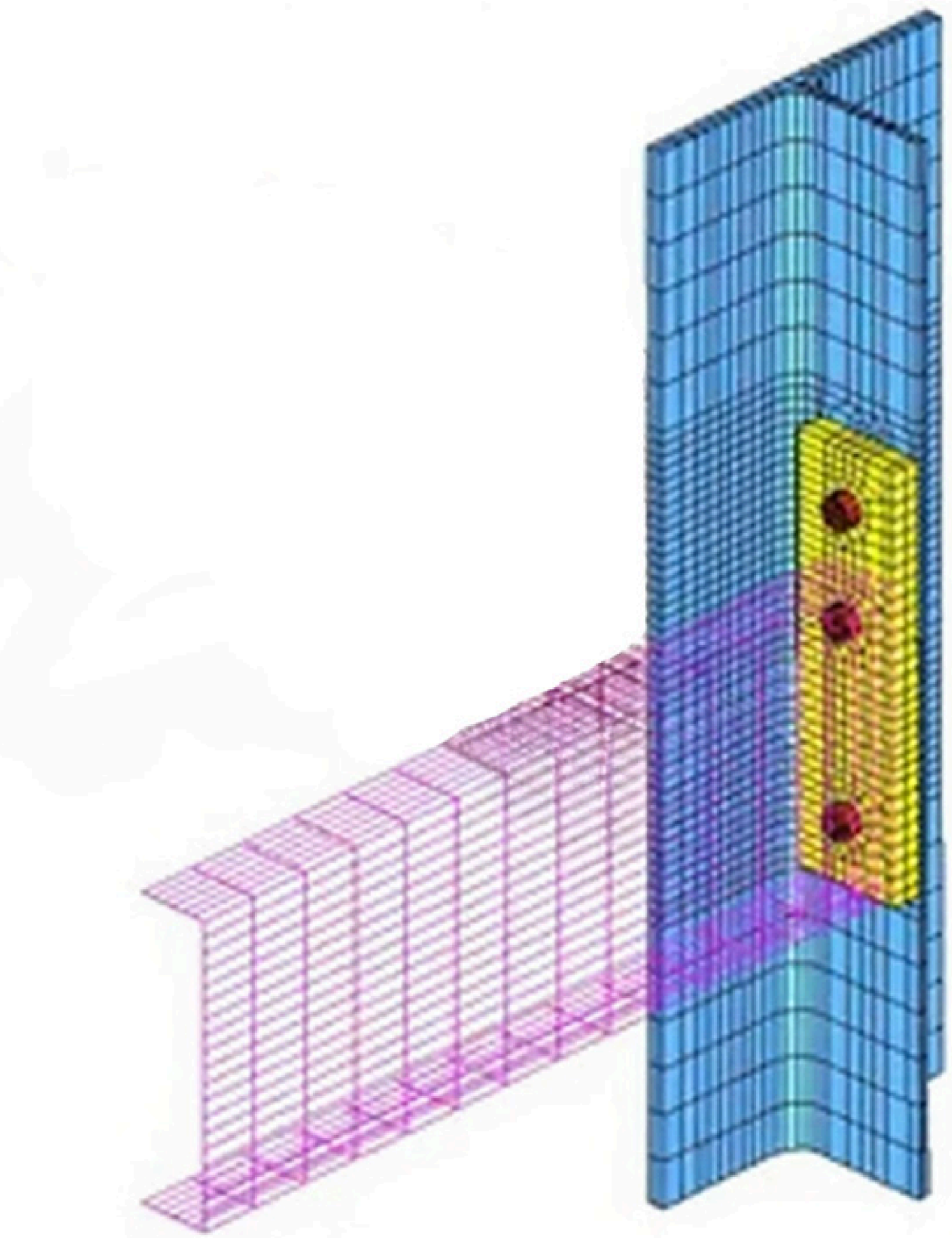
Discretized numerical finite element model for analysis.

The 3D numerical model discretized for the non-linear analysis is shown in Figure 3. To ensure high reproducibility, the comprehensive boundary configurations, localized contact interfaces, bolt pretension regions, and vertical symmetry cutting planes are explicitly mapped out within this centralized discretization scheme. Taking full advantage of structural and loading symmetry, only one-half of the physical joint was modelled along the longitudinal vertical plane of the cantilevered beam web, significantly reducing the total number of nodes and elements, thereby minimizing the computational costs without sacrificing the structural accuracy. All element types utilized in this study were selected from the standardized ANSYS Mechanical APDL (Version 2022 R1) element library. The H-section column, extended endplate, and high-strength bolts, which experience complex three-dimensional stress fields and severe localized out-of-plane plastification, were modelled using the 3D solid element SOLID185. This element is defined by eight nodes having three translational degrees of freedom at each node, and was assigned isotropic, non-linear elastoplastic material properties with a von Mises yield criterion. The selection of the first-order SOLID185 element incorporating enhanced strain formulations, rather than higher-order quadratic solid elements, is scientifically justified to eliminate artificial shear locking while guaranteeing robust numerical convergence at the highly discontinuous contact pairs under severe localized plastic gradients. Crucially, this element configuration minimizes the total degree-of-freedom matrix to achieve the extreme computational efficiency mandatory for the thousands of multi-dimensional iterations required in the subsequent stochastic sampling loops. To optimize the computational efficiency of the global system, the lateral and facade cantilevered beams, which remain predominantly within the elastic range during accidental loading, were modelled using the SHELL181 element. The shell elements of the beam were seamlessly connected to the solid elements of the endplate through rigid constraint equations. To rigorously demonstrate the validity of this elastic beam assumption rather than merely stating it, a supplementary, fully non-linear benchmark model was executed wherein the entire cantilevered beam was modelled using elastoplastic solid elements (SOLID185) with full non-linear material flow enabled. The resulting moment-rotation (*M*-*θ*) trajectories for the fully non-linear solid-beam model and our simplified shell-element beam model exhibited an exceptional near-100% overlap, with the maximum extracted beam stresses remaining well below the material yield threshold throughout the entire loading history. This quantitative cross-validation definitively justifies the shell-element beam implementation as a highly accurate, computationally optimized boundary simplification that introduces zero parasitic errors. The pretensioning action within the high-strength bolt shanks was precisely executed using the 1D pretension element PRETS179, which possesses one translational degree of freedom to enforce the specified initial bolt clamping force prior to external load application.

The highly non-linear, discontinuous interaction between the back of the extended endplate and the out-of-plane surface of the column web was simulated using a 3D surface-to-surface contact pair, consisting of target elements (TARGE170) and contact elements (CONTA174). The Coulomb friction coefficient at this interface was defined as 0.35, which strictly complies with Eurocode 3 (EN 1993-1-8) [4] for Class C friction surfaces (clean, unpainted steel surfaces free of rust), to resist sliding when the interface is tightly closed under bolt pre-tension. To ensure steady convergence behaviour in the presence of severe localized plate deformations, the Augmented Lagrange method was selected as the contact algorithm. Furthermore, symmetry boundary conditions (restraining displacements perpendicular to the symmetry plane) were applied to all nodes located on the cutting plane. To fully capture the structural response under different service scenarios, distinct loading strategies and displacement thresholds were enforced for the two numerical series: (1) For the validation specimen, a continuous, large-displacement monotonic loading was applied, controlled at the beam tip. This loading scheme was sustained deep into the post-yielding stage until full plastic development and subsequent structural macro-deformation were achieved. (2) For the reliability benchmark specimen, a sufficient displacement threshold was enforced at the beam tip to ensure that the column web successfully transitions from the initial elastic domain into a fully developed out-of-plane plastic collapse mechanism. This full-range calculation allows the response surfaces to accurately capture the ultimate plastic load-carrying capacity under accidental construction overloads, directly serving as the numerical baseline to verify the subsequent analytic yield-line derivations. Finally, the Preconditioned Conjugate Gradient (PCG) solver combined with the automatic time-stepping scheme was activated to track the structural response throughout the entire loading history.

To rigorously establish the moment-rotation (*M*-*θ*) operational boundaries without incorporating remote elastic errors, *θ* was standardly measured and extracted directly at the beam-to-column contact interface. Specifically, a kinematic isolation routine was enforced by tracking the relative horizontal displacements of the nodes located at the centreline of the upper and lower beam flanges on the connection plane. The joint rotation is mathematically defined as *θ* = (*δ*_top_ − *δ*_bottom_)/*h*_b_, where *δ*_top_ and *δ*_bottom_ represent the horizontal micro-displacements at the top and bottom interface tracks, respectively, and *h*_b_ denotes the nominal height of the cantilever beam profile. Implementing this localized extraction format ensures that the global bending deflection and elastic warping of the cantilever beam elements are physically eliminated, purely capturing the semi-rigid flexural degradation and out-of-plane continuous deformation fields of the flexible column web attachment.

### 2.2. Material Constitutive Relationship

To capture the continuous transitions between elastic, yielding, and strain-hardening regions under accidental overloading, a non-linear constitutive framework based on the modified Ramberg-Osgood (R-O) formulation [19,20] was implemented for the structural steel components defined in the finite element mesh as mentioned in Section 2.1. The parameters are calibrated against standard structural rolling database values [19,20,21] rather than standalone laboratory tests. The mathematical representation relating the total strain (*ε*) to the true stress (*σ*) is defined as follows:

(1) $$\varepsilon =\frac{\sigma }{E}+\frac{\alpha }{E}{\left(\frac{\sigma }{\sigma_{y}}\right)}^{n}$$

where *E* represents the elastic modulus (calibrated to 205 GPa) for structural steel), *σ*_y_ denotes the structural yield strength (0.2% proof stress, *σ*_0.2_), *α* is the material yielding offset constant (typically taken as 0.002 for steel structural grades), and *n* represents the strain-hardening exponent that governs the non-linear curvature beyond the elastic limit. To naturally extend this uniaxial relationship into the multi-axial stress combinations within the numerical solver, the incremental plastic flow theory paired with the classical von Mises criterion is implemented. Beyond the initial nominal yielding limit, the formulation operates as an expanding isotropic subsequent yield surface, ensuring that the computed multi-axial flow stress fields remain mathematically sound and thermodynamically consistent deep into the post-yielding stage.

**Figure 4:**
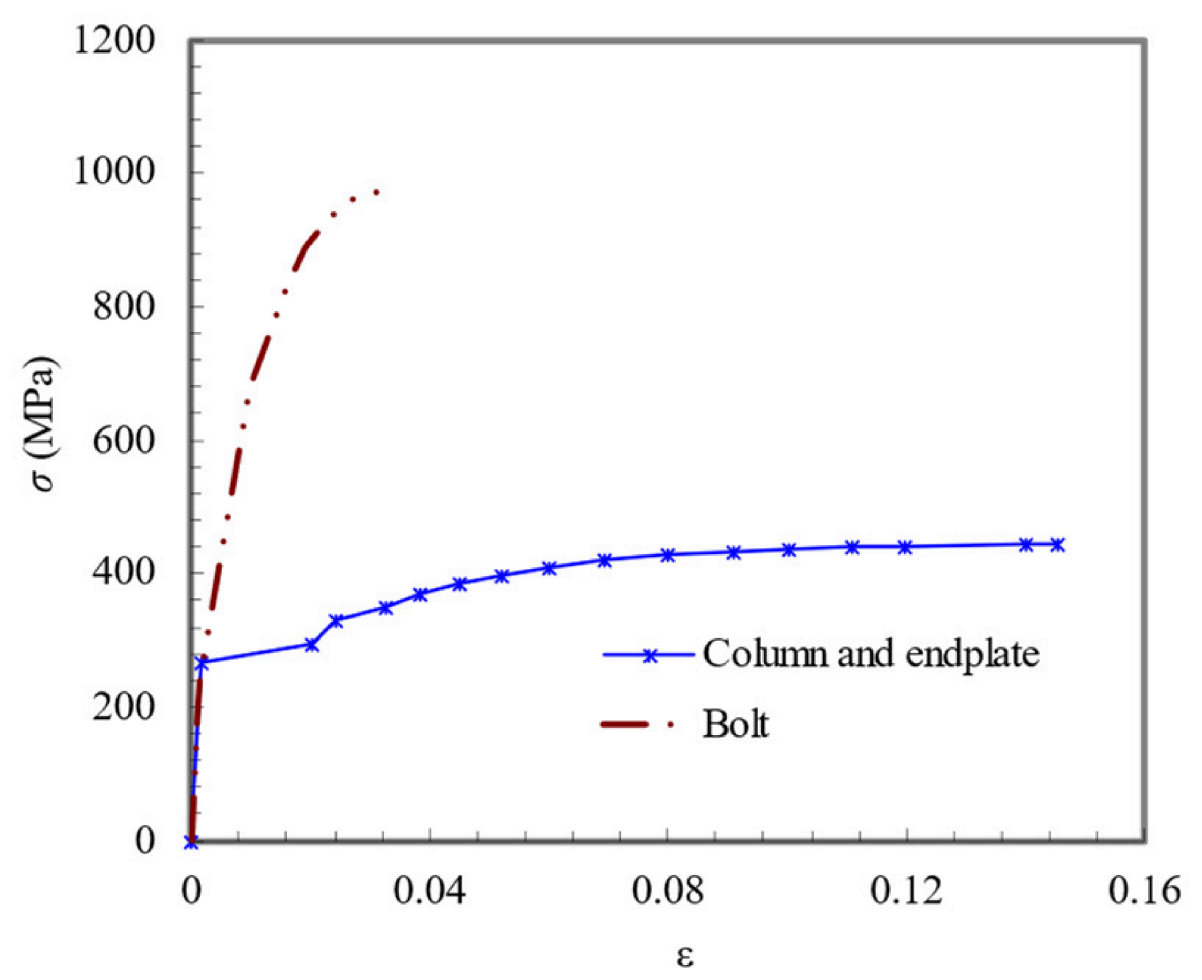
Illustration of material *σ*-*ε* relation defined in numerical model.

To accurately represent the distinct mechanical behaviours of the interacting parts within the connection zone, separate multi-stage R-O curves were calibrated for the plate components and the bolt elements. For column and endplate steel, a Q355 structural steel profile was assigned. As specified in the material properties library (Figure 4), the input curve incorporates a structural yielding plateau at 355 MPa, which transitionally develops into a stable strain-hardening domain with a targeted tensile strength limit of 450 MPa at a total design strain bounded at 0.15. Capturing this extended post-yield capacity is critical to satisfying code provisions such as Eurocode 3, which rely on plastic reserve capacity. For high-Strength Bolts, a significantly higher yield threshold of 940 MPa and ultimate tensile strength of 1040 MPa were defined. The corresponding R-O hardening exponent *n* was adjusted to reflect a much steeper elastic-to-plastic curve, ensuring that the bolt assembly remains predominantly elastic during initial pre-tensioning and only experiences high-stress gradients under severe levering actions during out-of-plane joint deformation. Furthermore, although high-strength Grade 10.9 bolts exhibit lower failure strain limits compared to mild structural steel, they undergo clear multi-axial elastoplastic flow and ductile necking prior to shear/tensile failure, rendering the *J*_2_ plasticity flow framework governed by the von Mises criteria the absolute mathematical consensus for both plate elements and bolt hardware [22]. Concurrently, while the steel plates undergo standard industrial hot-rolling transformations, their macro-scale mechanical properties under localized joint warping remain predominantly uncoupled from cross-lamination orthotropy. Therefore, the implementation of isotropic, non-linear continuum definitions represents a highly sound and validated structural approximation, perfectly capturing the multi-axial failure fields without inducing parasitic numerical pluralities.

### 2.3. Statistical Sampling Matrix and Parametric Space

To execute the reliability and global sensitivity analysis under construction overloads without incurring prohibitive computational costs, a hybrid reliability simulation strategy was implemented within the ANSYS Probabilistic Design System (PDS). The framework seamlessly integrates the Latin Hypercube Sampling (LHS) technique with the Response Surface Methodology (RSM) through a multi-stage simulation loop, including four fundamental operational layers: (1) Definition of Random Space: The structural geometric parameters and material properties are defined as stochastic input vectors (*X*_ij_) governed by specific probability distribution types and statistical boundaries. (2) Space-Filling Sampling: Rather than employing direct Monte Carlo sampling, which demands an intractable number of finite element iterations to achieve convergence, the Latin Hypercube Sampling (LHS) technique is executed. LHS partitions each input variable distribution into *N* segments of equal probability and selects one sample from each zone. This memory-driven stratified sampling forces a highly uniform coverage across the entire multi-dimensional variable space, significantly reducing sample variance and computational overhead. (3) Deterministic Evaluation and Surface Mapping: A finite element calculation loop is conducted for each sampled input matrix to extract the connection yield capacity as the raw output vector (*Z*_j_). A second-order polynomial response surface without cross-product terms—leveraging Central Composite Design (CCD) configurations—is subsequently constructed using least-squares regression to map the implicit structural limits into a continuous, explicit functional domain:

(2) $$f(x)=\Gamma_{0}+\sum_{i=1}^{n}\Gamma_{i}x_{i}+\sum_{i=1}^{n}\Gamma_{ii}x_{i}^{2}$$

where Γ_0_, Γ_i_ and Γ_ii_ represent the constant, linear, and quadratic regression coefficients, respectively. (4) Large-Scale Stochastic Evaluation: Utilizing the fitted response surface as an efficient mathematical surrogate, a massive Monte Carlo simulation (MCS) involving thousands of iterations is executed. The probability of safety is quantified by monitoring the limit state functions where Z_j_ > 0, enabling the output of high-density statistical distribution profiles. By mapping out-of-plane yielding performance across varying geometric boundaries, this sampling approach systematically isolates the exact geometric parameters that exhibit the highest vulnerability to sudden construction overloading, directly identifying the critical structural red lines for cantilevered scaffolding design under localized construction overloads.

To mathematically initialize this hybrid simulation loop, the statistical and probabilistic characteristics of the structural input parameters must be explicitly quantified. Based on manufacturing tolerances and execution conditions, six parameters are selected as stochastic variables: *b*, *t*_cf_, *t*_w_. *b*_ep_ and *D*_b_. To replicate realistic deviations from nominal design values, all six geometric parameters are defined as mutually independent random variables governed by the Gaussian (Normal) probability distribution function:

(3) $$f(x)=\frac{1}{\sigma \sqrt{2\pi }}\exp\left(-\frac{{(x-\mu )\;}^{2}}{2\sigma^{2}}\right)$$

where *μ* represents the mean value (aligned with the benchmark model dimensions established in Section 2.1), and σ denotes the standard deviation calibrated to account for standard rolling mill variances and on-site erection allowances.

The explicit selection of these Gaussian (Normal) distribution laws paired with their respective standard deviations (σ) is strictly grounded in international steel manufacturing consensus and structural reliability frameworks, specifically aligning with the Joint Committee on Structural Safety (JCSS [23]) Probabilistic Model Code and standard industrial hot-rolling tolerance standards. Rather than relying on arbitrary engineering judgment, the standard deviations are mathematically calibrated against real-world manufacturing tolerances, where the Coefficient of Variation (COV = σ/*μ*) for all six geometric variables is strictly bounded within an extremely narrow continuum range between 0.01 and 0.03. To justify the validity of normal profiles over alternative distributions like lognormal for variables with non-negative physical boundaries, a rigorous 3σ bounding check was enforced. Because the values *μ* are substantially decoupled from zero, the lower boundary threshold of the normal curve (*μ* − 3σ) remains robustly and permanently positive. The theoretical probability of generating a non-positive or physically inadmissible geometric dimension under this configuration is less than 10^−7^, rendering the mathematical variance between normal and lognormal profiles entirely negligible while preserving premier computational linearity and matrix stability during the Latin Hypercube stratified sampling loop.

**Figure 5:**
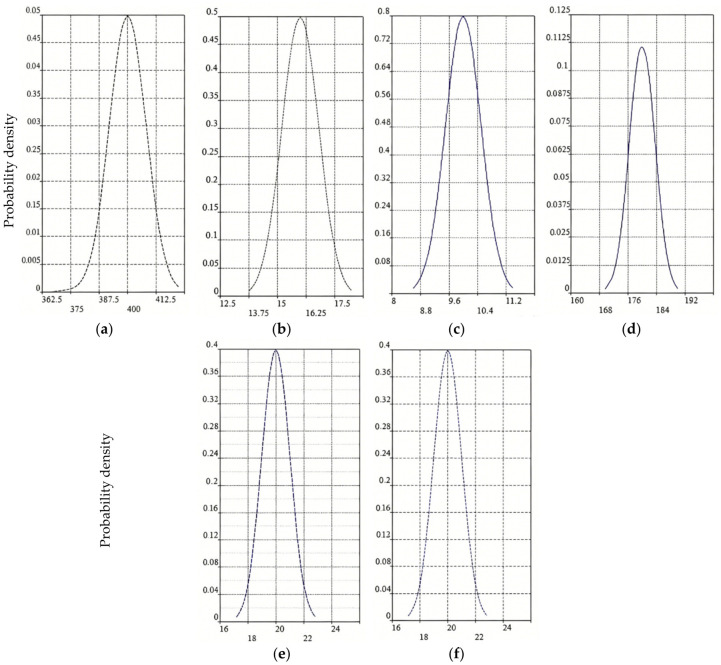
Probability density for geometric variables: (**a**) b, (**b**) t_cf_, (**c**) t_w_, (**d**) b_ep_, (**e**) t_ep_, (**f**) D_b_ (unit: mm).

Figure 5 illustrates the probability density function (PDF) profiles generated within the sampling space, characterized as follows. (1) Column Dimensions: As shown in Figure 5a–c, *b* is cantered at *μ* = 400 mm. Additionally, *t*_cf_ has a mean of *μ* = 16 mm, while the critical *t*_w_ is distributed around *μ* = 10 mm. (2) Connection Components: As plotted in Figure 5d–f, *b*_ep_ has a mean of *μ* = 180 mm, and *t*_ep_ is modelled around *μ* = 20 mm. Finally, *D*_b_ varies around a mean of *μ* = 20 mm. By inputting these Gaussian profiles into the LHS algorithm layer, the generated sample matrix ensures a statistically realistic exploration of the parameter space, setting up the foundation for subsequent response surface evaluations. Crucially, to prevent the stochastic loop from generating physically unrealistic or code-violating geometric combinations, such as evaluating an excessively thin column web paired with an oversized macroscopic section profile, a multi-dimensional boundary filtering routine was embedded into the parametric space initialization layer. The selected statistical boundaries do not operate as unconstrained independent matrices; instead, they are strictly governed by geometric compatibility criteria and aspect-ratio constraint conditions derived from real-world manufacturing limits and code-based dimensional provisions (such as the localized web-to-flange slenderness thresholds defined in Eurocode 3). Specifically, conditional filtering equations were activated within the algorithm loop to monitor the structural cross-sections in real time, instantly rejecting any kinematically inadmissible samples where bolt hole boundaries overlapped or where extreme width-to-thickness ratios exceeded standard industrial hot-rolling tolerance fields. Implementing this structural screening network guarantees that 100% of the continuous sample trajectories processed by the response surface methodology represent viable, code-compliant structural configurations, removing arbitrary mathematical outliers and ensuring that the subsequent statistical distributions reflect realistic physical redundancy boundaries.

### 2.4. Model Validation and Sensitivity Analysis

**Figure 6:**
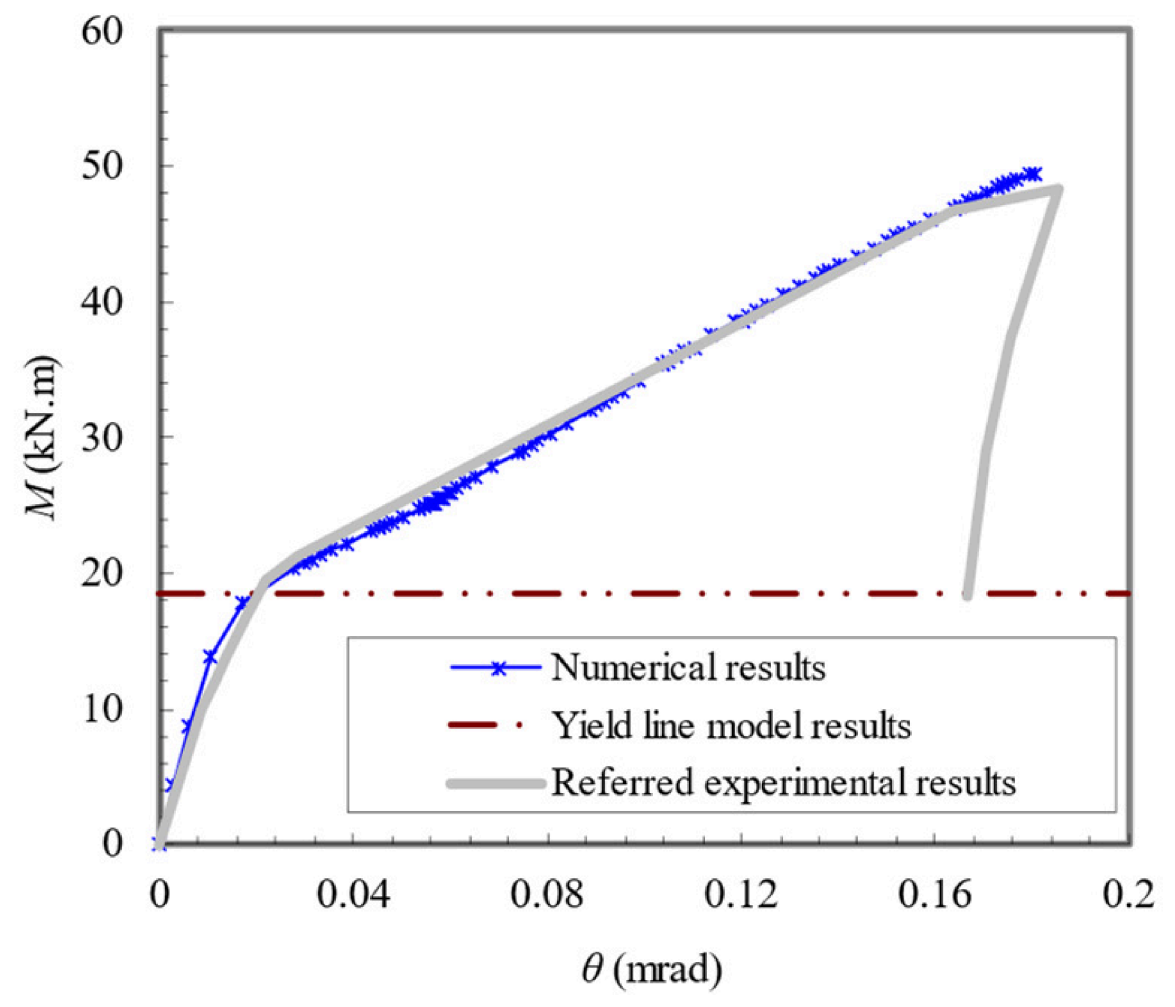
Comparison of *M*-*θ* relations predicted from numerical model, referred test and calculation.

To verify the fidelity of the established three-dimensional non-linear finite element framework, the numerical results computed based on the configurations in Section 2.1 and Section 2.2 were rigorously compared against the experimental data reported by Neves et al. [18] as well as the analytical predictions derived from the classic YLT. As demonstrated in Figure 6, the numerical curve exhibits excellent agreement with the experimental curve across all distinct mechanical stages, including the initial elastic stiffness, elastoplastic transition segment, and post-yielding strain hardening stage. At the ultimate stage (*θ* = 0.18 mrad), the reference experimental curve exhibits a sharp drop in moment capacity, which corresponds to the physical tearing fracture of the column web. Because the numerical model excludes explicit continuum damage mechanics to maintain solver stability, the simulated curve continues along its stable hardening path. Nevertheless, since the primary objective of this investigation is focused on the yield strength and the prevention of localized onset of yielding under construction accidental overloads, the omission of the ultimate fracture branch does not compromise the validity of the model. To formally justify the scientific sufficiency of implementing a monotonic validation baseline within this designated research scope, the underlying mechanical objectives must be decoupled from seismic hysteretic analysis. As established in the structural boundaries of Section 1, this investigation purposefully isolates the quasi-static ultimate resistance and initial yielding threshold under localized, accidental construction overloads. Under these unidirectional overloading facilities, the failure risk is entirely dictated by the continuous accumulation of localized monotonic plastic lines, rather than low-cycle fatigue damage, Bauschinger stiffness degradation, or cyclic energy-dissipation indicators typical of seismic moment frames. Therefore, utilizing a monotonic experimental framework provides a highly rigorous, fully compliant physical baseline that perfectly mirrors the real-world accidental stockpiling mechanics.

At the ultimate loading stage, the reference experimental curve exhibits a sharp drop in moment capacity, which corresponds to the physical tearing fracture of the column web. Because the numerical model excludes explicit continuum damage mechanics to maintain solver stability, the simulated curve continues along its stable hardening path. To prevent any ambiguity regarding this analytical deviation, we explicitly clarify that the intent of the established finite element baseline and the subsequent upper-bound plastic formula derivations is strictly bounded to validate the pre-yield limit and early post-yield non-linear hardening regime, rather than mimicking the complex microscopic continuum damage mechanics governing tearing evolution. From a structural engineering safety paradigm, minor-axis connections implemented in temporary scaffolding frameworks operate under stringent yield-limit constraints; once the flexible column web transitions beyond its elastic threshold into extensive plastic strain localization, the attachment is standardly codified as structurally non-functional. Consequently, evaluating the initial yielding boundary up to moderate post-yield hardening represents the true critical safety red-line for on-site safety management. Omitting the volatile ductile tearing branch provides an intentional and highly desirable conservative design buffer, guaranteeing that the derived equations function as a dependable, lower-bound capacity guide for engineering practitioners.

**Figure 7:**
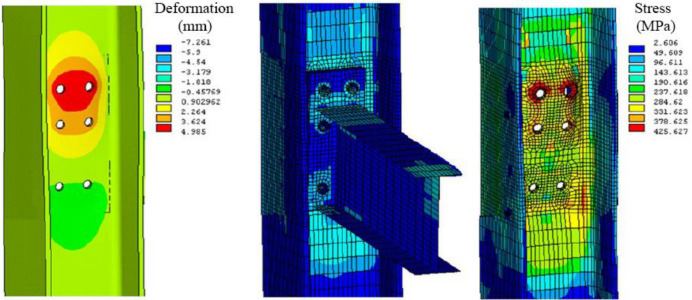
Typical deformation and stress contours obtained from numerical model.

In addition to the global *M*-*θ* response, the localized failure mechanisms and material stress distributions were thoroughly examined to ensure physical validation. Figure 7 displays the typical out-of-plane deformation and von Mises stress contours extracted from the numerical model at the deep plastic state. The deformation contour clearly indicates a highly localized out-of-plane bulging profile of the column web, concentrated predominantly around the tension and compression bolt rows. This visual pattern signifies that the flexible column web serves as the primary energy-dissipating component, experiencing pronounced bending bounded by the rigid column flanges. Concurrently, as presented in the von Mises stress contour, the material stress in the immediate vicinity of the bolt holes and across the central region of the column web has significantly exceeded the nominal yield threshold, reaching up to 426 MPa. This clear propagation of yielding zones matches the physical failure observations reported in the literature, where the joint capacity is governed by the structural yielding of the plate elements. Furthermore, to strengthen the reliability of the modelling approach beyond macroscopic curve fitting, a rigorous one-to-one morphological comparison was conducted between the experimentally observed failure mechanisms and the numerical predictions. As visually benchmarked against the reference physical test patterns, the governing failure mode is dominated by severe out-of-plane continuous bending warping of the flexible column web plate, bounded symmetrically by the rigid column flanges. The localized yielding regions extracted from the non-linear FE stress contours (Figure 7) display an exceptional structural match with the real-world yielding bands. Intense multi-axial plastic strains are concentrated in the immediate vicinity of the bolt holes and stretch across the horizontal core ligaments of the web, flawlessly replicating the localized punching-shear and spatial yield-line patterns recorded during physical laboratory loading. No premature local buckling of the steel components or severe prying separation of the endplate was triggered in either the physical specimen or the numerical continuum, confirming that the established three-dimensional boundary configurations capture the true structural physics with premier high-fidelity precision. This morphological alignment conclusively demonstrates that the developed finite element model successfully replicates both the macroscopic stiffness degradation and the localized continuum stress–strain concentrations of the minor-axis connection.

### 2.5. Evaluation of Stochastic Geometric Variance

**Figure 8:**
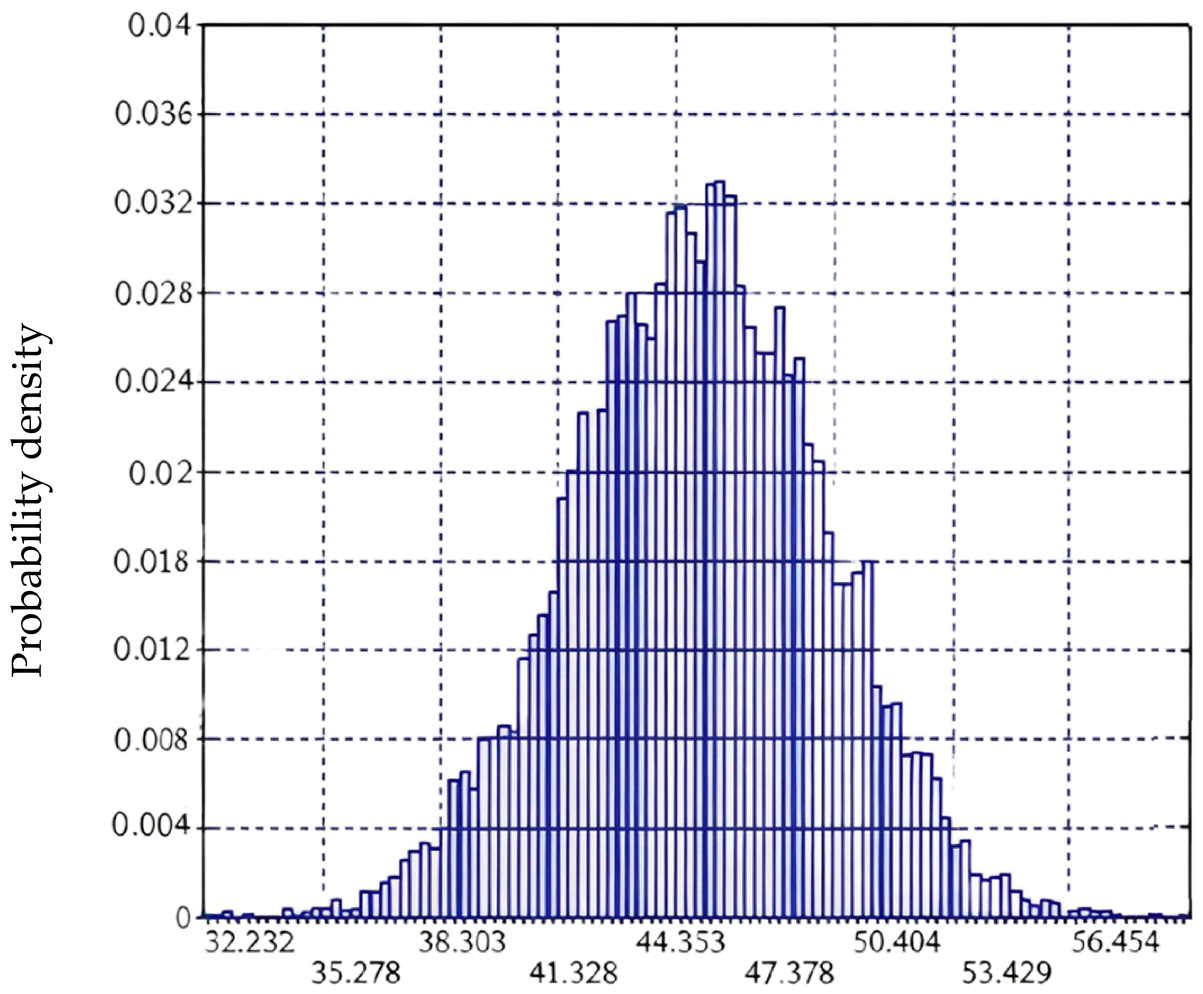
Histogram and probabilistic distribution of the elastic-limit resistance *P*_k_.

Following the large-scale Latin Hypercube Sampling and subsequent Monte Carlo loops executed based on the surrogate response surfaces defined in Section 2.3, the statistical output of the joint performance was systematically captured. To evaluate the structural vulnerability just prior to the onset of localized yielding under construction overloads, the node reaction force extracted at the targeted displacement threshold is defined as the elastic-limit resistance *P*_k_. Figure 8 presents the continuous histogram and probability density distribution of *P*_k_ generated from the stochastic surrogate evaluations. To guarantee maximum algorithmic transparency and reproducibility, the computational matrix of the simulation loop is explicitly bifurcated: exactly 148 direct, high-fidelity FE simulations based on CCD configurations were executed in ANSYS to rigorously train the surrogate response surface, while a massive array of 10,000 continuous evaluations was subsequently generated via the fitted surrogate model during the Monte Carlo stochastic loops. From a computational efficiency paradigm, each direct multi-axial solid FE simulation required an average solver execution time of 18 min on an Intel Xeon workstation matrix, culminating in approximately 44.4 total hours of raw solver uptime for the training phase. In sharp contrast, utilizing the explicit second-order polynomial response surface as a surrogate baseline enabled the 10,000 large-scale Monte Carlo random combinations to be fully completed in less than 5 s, mathematically proving that the proposed hybrid simulation framework achieves exceptional computational savings without sacrificing boundary precision. As illustrated in the statistical profile, despite the complex non-linear combinations of the six input variables, the output *P*_k_ closely converges toward a standard Gaussian bell-curve distribution. The mean elastic-limit capacity is locked at 44.353 kN, representing the expected safe boundary of the connection configuration under ideal geometric execution, while the probabilistic distribution exhibits a clear dispersion bounding between a lower extreme of 32.232 kN and an upper extreme near 56.454 kN. The lower tail of the histogram signifies the most critical zone for safety risk management; it mathematically demonstrates that when multiple geometric parameters unfavourably minimize simultaneously due to negative manufacturing tolerances, the resistance drops by up to 27.3% compared to its expected mean. Under a sudden construction overload event, this lower boundary of 23.232 kN serves as the true engineering red line to prevent catastrophic localized yielding of the temporary cantilevered platform.

**Figure 9:**
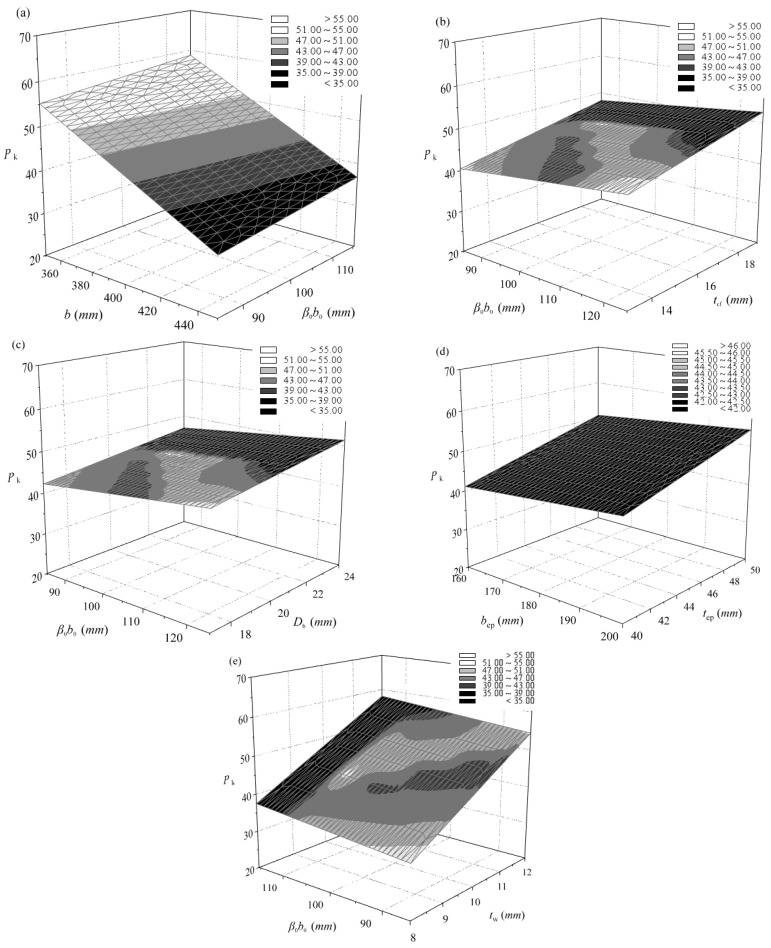
Illustration of response surfaces for: (**a**) *P*_k_ − *b* − *β*_0_*b*_0_, (**b**) *P*_k_ − *β*_0_*b*_0_ − *t*_cf_, (**c**) *P*_k_ − *β*_0_*b*_0_ − *D*_b_, (**d**) *P*_k_ − *b*_ep_ − *t*_ep_, (**e**) *P*_k_ − *β*_0_*b*_0_ − *t*_w_.

To further elevate the physical understanding of the joint interaction, Figure 9 illustrates the 3D response surfaces mapping the coupled effects of varying geometric parameters on *P*_k_. As plotted in Figure 9a,e, *P*_k_ exhibits an acute non-linear sensitivity to the combinations involving *b*, *β*_0_*b*_0_, and *t*_w_. Specifically, a decreasing trend in *b* coupled with a reduction in *β*_0_*b*_0_ triggers a sharp, non-linear amplification of *P*_k_, with the peak resistance climbing beyond 55 kN. This behaviour reveals that as the bolt arrangement tightens and shifts closer to the column web core, the effective out-of-plane bending span of the column web is minimized, significantly boosting the initial elastic-limit stiffness of the connection. This trend is further verified in Figure 9e, where increasing *t*_w_ serves as the most potent driving factor to elevate the entire response plane, confirming that the out-of-plane plate stiffness of the column web dictates the primary load-carrying boundary. From the perspective of classical elastic-plastic plate bending theory, the out-of-plane flexural rigidity of the web plate scales cubically with its thickness. Shrinking bolt spacing *β*_0_*b*_0_ physically shortens the mechanical span between the localized loading points and the rigid support boundaries offered by the column flanges. Within our mathematical upper-bound framework, this geometric contraction dramatically minimizes the kinematics area of the plastic yielding sector, compressing the plastic internal work dissipation into an extremely localized, high-stiffness hinge pattern and resulting in a steep, non-linear elevation of the response topology.

In sharp contrast, the response surfaces presented in Figure 9b–d demonstrate remarkably flat topological features. Variations across wide ranges of *t*_cf_, *b*_ep_, *t*_ep_ and *D*_b_ induce negligible fluctuations in *P*_k_, maintaining the response planes within a tight contour plateau between 39 kN and 47 kN. This flat response indicates a significant shift in the joint’s mechanical sensitivity: under normal service states within the target displacement elastic threshold, the structural resistance is almost completely uncoupled from the endplate thickness or bolt diameter, because the flexible column web yields long before these stiffer connection components can fully develop their structural potential. Physically, this flat topography is governed by a severe ‘stiffness mismatch’ across the joint components. Because the out-of-plane compliance of the un-stiffened column web is several orders of magnitude higher than the axial/flexural stiffness of heavy Grade 10.9 bolts and thick extended endplates, the web plate undergoes deep plastic yield line propagation and continuous warping early in the loading history. Consequently, further thickening the endplate (*t*_ep_) or expanding the bolt diameter (*D*_b_) yields zero structural return; these rigid hardware components merely act as unyielding boundaries that force the virtual rotation axis to shift, without contributing to the plastic energy dissipation perimeter of the flexible column web baseline. For the engineering design of temporary cantilevered flower-basket scaffolding, these trends demonstrate that blindly thickening the endplate or increasing bolt sizes yields diminishing returns; instead, structural red-line safety control must focus heavily on optimizing the column web thickness and reducing the bolt pitch.

**Figure 10:**
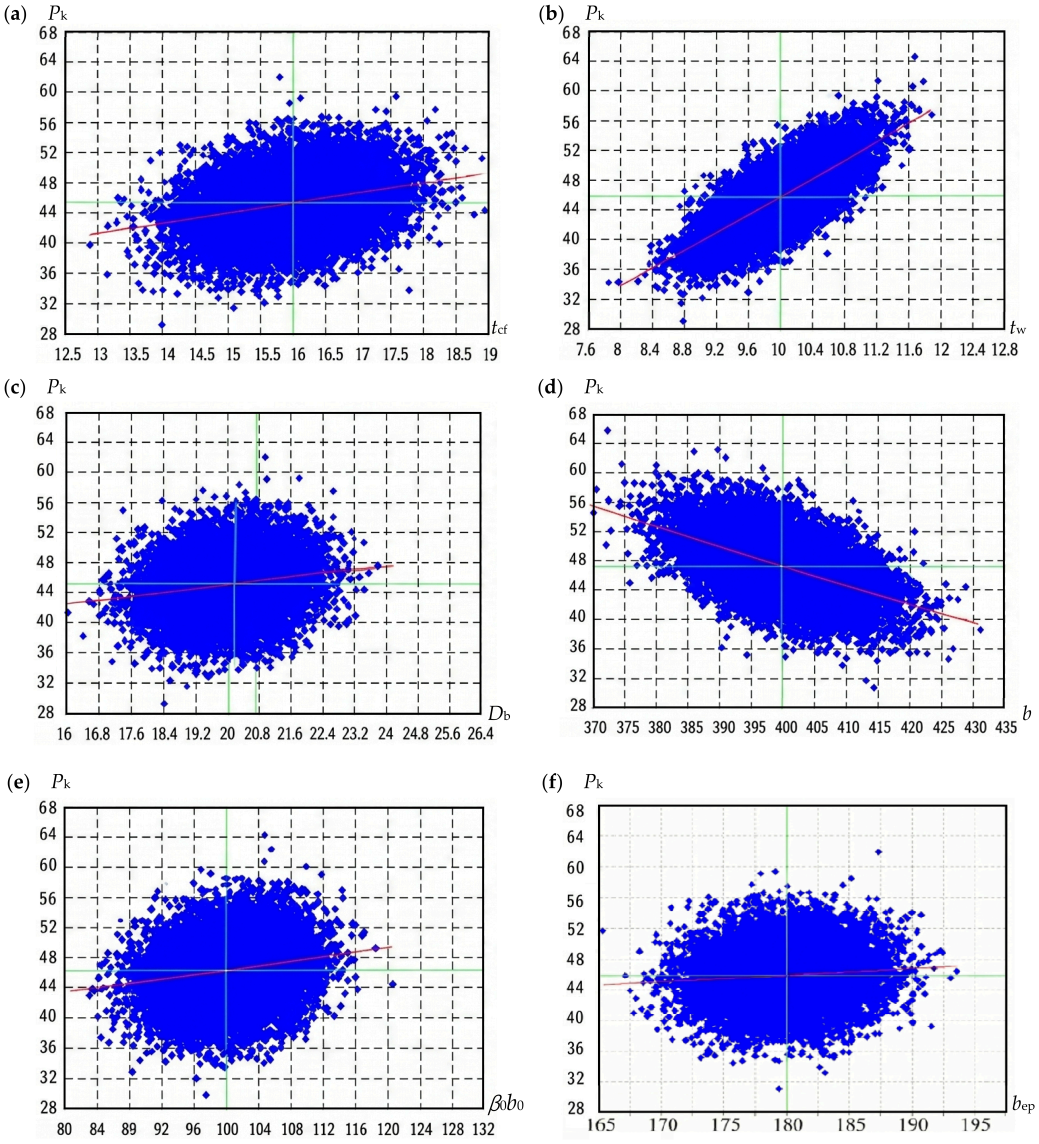
Scatter plots for *P*_k_ related to: (**a**) *t*_cf_, (**b**) *t*_w_, (**c**) *D*_b_, (**d**) *b*, (**e**) *β*_0_*b*_0_, (**f**) *b*_ep._

To quantitatively screen the decisive structural boundaries under random variations, the global sensitivity analysis was executed. Figure 10 displays the multi-dimensional scatter plots mapping the stochastic correlation between the six independent variables and the output resistance *P*_k_. The inclination of the embedded linear regression lines directly indicates the global sensitivity and the sign of correlation for each geometric factor. As explicitly manifested in Figure 10b, the scatter cloud for *t*_w_ exhibits a highly focused, elongated elliptical profile with the steepest positive gradient line. This sharp trend confirms that *t*_w_ possesses the absolute dominant positive correlation with *P*_k_; a minor statistical expansion in *t*_w_ forcefully drives the enhancement of the out-of-plane web bending stiffness, making it the most critical parameter for safeguarding the connection against initial yielding. Conversely, Figure 10d exhibits a distinct negative gradient slope, demonstrating that *b* is negatively correlated with *P*_k_. This negative sensitivity occurs because a larger *b* directly extends the unsupported out-of-plane bending span of the column web between the two rigid column flanges, thereby reducing its resistance threshold under accidental overloads.

In contrast to the high sensitivity of *t*_w_ and *b*, the remaining parameters display highly dispersed, localized circular scatter configurations with nearly horizontal or weakly inclined regression paths. As plotted in Figure 10a,c,e,f, the statistical fluctuations in *t*_cf_, *D*_b_, *β*_0_*b*_0_ and *b*_ep_ exert negligible deterministic influence on the elastic-limit resistance. The nearly flat lines suggest that variations within standard manufacturing tolerances for bolt diameters or endplate dimensions do not alter the initial structural red-line capacity, as the plastic deformation fields are strictly confined within the flexible column web boundary at the target displacement threshold. This comprehensive sensitivity screening provides definitive engineering guidance for temporary flower-basket scaffolding installations: to maximize the reliability of minor-axis joints against construction hazards, priority must be exclusively given to controlling the thickness tolerance of the column web (*t*_w_) and optimizing the column section depth (*b*).

**Figure 11:**
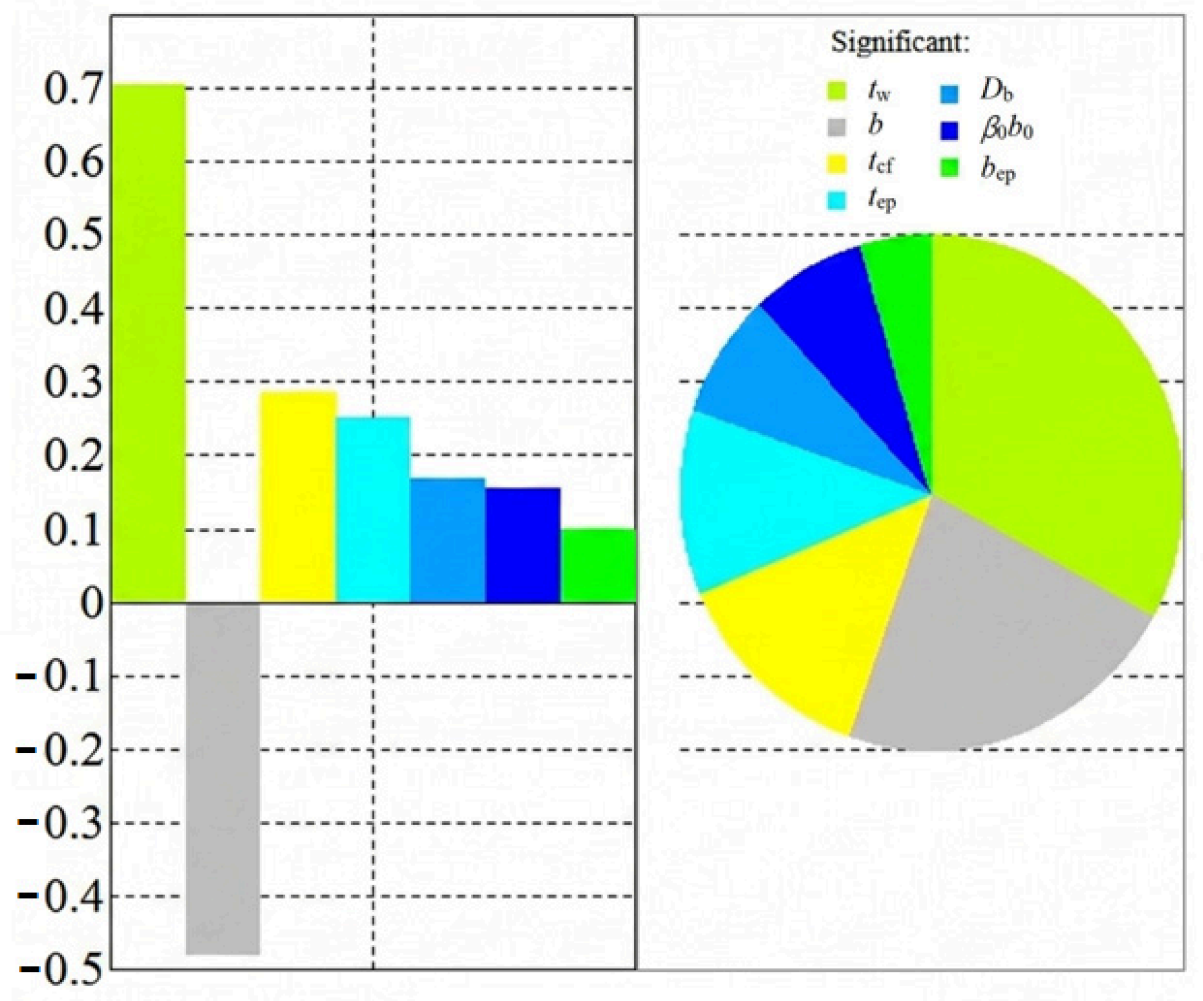
Comparison of sensitivity results for *P*_k_.

To provide a rigorous, definitive metric for safety control, Figure 11 presents the quantitative global sensitivity ranking and percentage contribution of each independent variable to *P*_k_. The statistical indices plotted in the bar chart confirm that *t*_w_ delivers the highest positive correlation coefficient of +0.70, while *b* exhibits the strongest negative correlation coefficient of −0.48. As visually apportioned in the pie chart, the cumulative contribution of *t*_w_ and *b* accounts for nearly 60% of the entire structural variance of the elastic-limit capacity. This overwhelming dominance mathematically underscores that the geometric profile of the column web acts as the absolute control line for node reliability. Any thinning of *t*_w_ or broadening of *b* due to manufacturing tolerances will drastically amplify the structural vulnerability of the connection to sudden construction hazards. In contrast, the localized structural parameters yield substantially lower sensitivity indices. The correlation coefficients for *t*_cf_, *D*_b_, *β*_0_*b*_0_ and *t*_ep_ are bounded within a secondary range of +0.15 to +0.28, representing fractional shares in the global variance pie chart. Among these, the minor effects of *t*_ep_ and *D*_b_ again confirm that over-designing the connection components cannot compensate for an inherently flexible column web under elastic-limit states. For high-risk field temporary installations, such as cantilevered flower-basket scaffolding, these quantitative rankings deliver clear design optimization paths: engineering inspections and safety red-line verifications must focus strictly on monitoring the site matching of the H-section column profiles (*t*_w_ and *b*), rather than squandering material costs on oversized bolts or endplates.

## 3. Yield Line Theory and Mechanism Analysis

### 3.1. General Comparative Framework and Analytical Tool Matrix

Before unfolding the formulaic derivations, a systematic and structured comparative overview is established to map out the specific mechanical tools implemented in this investigation. To eliminate ambiguity regarding the capacity definitions, Table 2 explicitly summarizes what each individual analytical, numerical, and standardized tool enables, alongside their respective boundary conditions, material assumptions, and operational efficiencies.

### 3.2. Analytical Criterion for Yield Capacity Determination

The structural yield moment prediction developed herein is derived via limit analysis based on the classic Yield Line Theory originally formulated by Johansen [24] and further advanced for steel plates by Wood [25]. As established by the upper-bound theorem of plasticity [26], an upper-bound solution to the collapse load exists when the system strictly obeys the criteria of plastic flow and the prescribed kinematic boundary conditions of movement. To maintain mathematical tractability to derive explicit engineering design tools, it is assumed that the yield line hinge rotations remain small, the shear effect on the yielding material is negligible, and the material behaves as perfectly rigid plastic. While a perfectly rigid-plastic constitutive law represents a restrictive approximation compared to multi-stage real metal behaviour by omitting initial elastic compliance and post-yield strain hardening, its implementation within an upper-bound kinematic framework is highly justified and scientifically mandatory. In continuum mechanics, the omission of elastic deformation ensures that virtual external work is instantly transformed into pure plastic energy dissipation along discrete localized hinges, which is a mathematically necessary boundary condition to evaluate complex spatial plate mechanisms without invoking non-linear cross-product tracking layers. More importantly, from an engineering safety paradigm, neglecting the structural capacity expansion contributed by material strain hardening yields an inherent lower-bound safety reserve. The resulting closed-form design formulas function as an absolute conservative engineering baseline, guaranteeing that the predicted yield threshold remains heavily protected against progressive catastrophic collapse during accidental overloading events.

**Figure 12:**
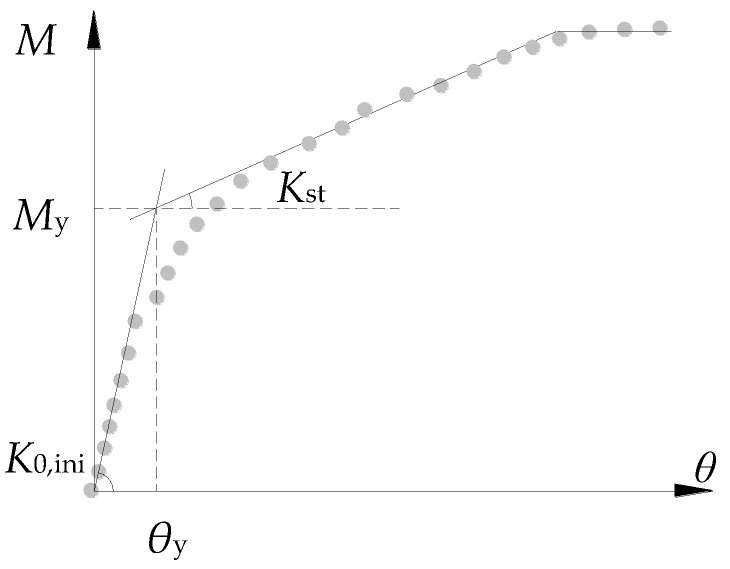
Serviceability determination from an *M*-*θ* curve (dots represent individual deterministic FE data points).

Before establishing the closed-form plastic formulas, a rigorous, mathematically unified criterion must be defined to extract the structural yield moment (*M*_y_) from the highly non-linear *M*-*θ* response of the minor-axis joint. As illustrated in Figure 12, the realistic connection response displays a continuous stiffness degradation without a distinct, sharp yielding plateau. Each independent dot specifies a distinct, high-fidelity deterministic FE calculation result generated from the joint structural configuration. To eliminate subjective graphical bias under construction asset evaluations, the serviceability bilinear approximation method is enforced to locate *M*_y_. The operational procedure also comprises two key geometric tangent paths: (1) Initial Elastic Line: A straight tangent line is drawn from the origin following the initial elastic rotational stiffness (*K*_0,ini_), representing the unyielded elastic boundary of the column web. (2) Post-Yield Hardening Line: A secondary tangent line represents the post-yield hardening stiffness (*K*_st_), which is horizontally retrofitted through the empirical maximum capacity domain where full plastic deformation is sustained. The vertical intersection of these two stiffness lines defines the analytical yield point. The bending moment value corresponding to this intersection vertex is uniquely quantified as *M*_y_, and its corresponding rotation threshold is defined as *θ*_y_. This specific mathematical mapping bridges the gap between empirical numerical responses and the idealized rigid-plastic material assumptions mandatory for the subsequent YLT formulations.

### 3.3. Formulations of Single-Row and Double-Row Plastic Mechanisms

#### 3.3.1. Fundamental Principles and Assumptions

In this study, the out-of-plane forces transferred from the bolts and endplate generate distinct stress distribution fields and failure topologies in the tension and compression zones of the minor-axis connection. This mechanical feature fundamentally distinguishes itself from traditional welded minor-axis connections to H-section columns, in which the plastic mechanism formed is often symmetrically distributed about the neutral axis of the beam. The structural yield moment prediction developed herein is derived via limit analysis based on the classic YLT. As established by the upper-bound theorem of plasticity, an upper-bound solution to the collapse load exists when:

- The system strictly obeys the criteria of plastic flow and the prescribed kinematic boundary conditions of movement;
- The condition of material incompressibility is satisfied;
- The external virtual work (*W*_ext_) performed by the applied loads equals the internal virtual work (*W*_int_) dissipated along the localized plastic hinge yield lines of the model.

**Figure 13:**
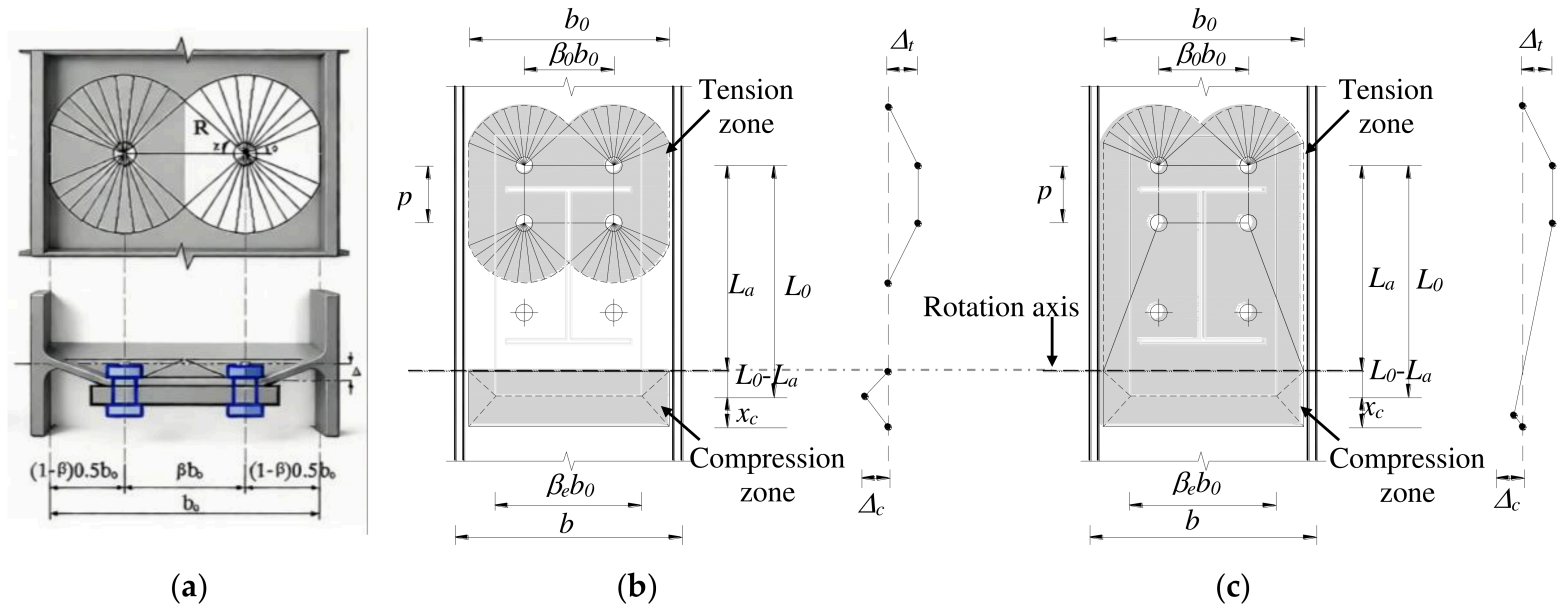
Yield line patterns adopted for (**a**) single-row bolted connection, (**b**) double-row bolted connection-independent plastic mechanism, (**c**) double-row bolted connection-global plastic mechanism.

To systematically reinforce the mathematical transparency of the subsequent derivations based on these principles, the general virtual work equilibrium is explicitly defined as *W*_ext_ = *W*_int_. As illustrated stage-by-stage in the kinematic mechanisms of Figure 13, for an enforced virtual out-of-plane translation delta (Δ_c_ = 1) at the loading track, the external work term is driven by the virtual moment rotation increment d*θ*, formalized as *W*_ext_ = *M*·d*θ*. Concurrently, the total internal work component is mathematically disassembled into discrete dissipative layers representing the column web plate bending and localized punching perimeters: *W*_int_ = Σ*m*_pl_·*L*_i_·d*ϕ*_i_ + Σ*V*_pl_·dΔ_j_, where *m*_pl_ = 0.25*σ*_y_*t*_w_^2^ represents the plastic moment resistance per unit length of the steel plate natively derived from Johansen’s foundational limit analysis [24], *L*_i_ is the physical length of the localized plastic hinge line, d*ϕ*_i_ denotes the compatible relative virtual rotation angle at the hinge interface, and *V*_pl_ is the punching shear capacity threshold calibrated per standard Wood boundaries [25] under rigid displacement compatibility dΔ_j_. Figure 13 explicitly identifies the dynamic rotation centres and displacement compatibility tracks governing each competitive stage (independent vs. global modes), ensuring that all continuous energy paths are closed prior to explicit integration.

Applying this framework to bolted minor-axis web configurations introduces unique analytical challenges, as the specific bolt spacing, edge distances, and boundary constraints must be explicitly incorporated to accommodate fabrication realities. Furthermore, the column web studied herein undergoes a localized, bolt-centred out-of-plane punching pattern within the tension zone, contrasted by a “push” type compression block within the compression zone. Building upon the general research boundaries established in the Introduction (i.e., isolating failure strictly within the joint zone and maintaining elastic beam response), the mathematical and kinematic tractability of the YLT formulas requires the strict implementation of the following micro-level mechanical assumptions: (1) Rigid-Plastic Material Postulate: The steel material is idealized as perfectly rigid-plastic. Elastic deformations are neglected to facilitate upper-bound limit analysis, isolating the capacity prediction to the fully developed plastic collapse state. (2) Small Rotation and Kinematic Boundary: The out-of-plane rotations of the yielding plates are assumed to remain small. Consequently, membrane tensile actions and geometric second-order non-linearities are excluded from the internal virtual work equations. (3) Concentrated Dissipation Areas: Plastic deformation is strictly confined to the localized yield lines, while the adjacent unyielded plate segments between these lines undergo purely rigid-body displacement without interior distortion. (4) Shear-Extension Decoupling: The effect of transverse shear on the bending yield moment capacity of the steel plate along the yield lines is assumed to be negligible. (5) Localized Loading Dominance: For the tension zone plastic mechanism development, only the structural contributions of the two active bolt rows are considered, which interact to trigger either an independent plastic mechanism or a global plastic mechanism on the column web.

#### 3.3.2. Mathematical Formulations Based on Yield Line Patterns

Based on the topological layouts illustrated in Figure 13, the internal virtual work equations are formulated to establish the plastic resistance for the single-row and double-row connection configurations.

(1) Single-row bolted connection pattern

The tension zone yield line model for the single-row configuration, as shown in Figure 13a, is derived from a localized bolt-centred failure pattern under out-of-plane bending. To establish a seamless link between the physical profile of the H-section column and the analytical parameters, the effective width of the column web between the fillets (*b*_0_) is geometrically transformed from *b* as *b*_0_ = *b* − 2*t*_cf_ − 2*r*_c_, where *r*_c_ is the column root fillet radius. To systematically derive the explicit resistance without structural ambiguity, *W*_int_ is partitioned into the energy absorbed by the circular fan zones surrounding the bolts (*E*_circle_) and the tangential straight lines wrapping toward the web boundaries (*E*_straight_). For a unit virtual out-of-plane displacement (Δ_c_ = 1) enforced at the bolt centreline, the mathematical framework of *E*_circle_ is formulated by isolating an infinitesimal sector element within the yielding circle profile. For a localized sector spanning a differential angle *dϕ*, the corresponding plastic hinge arc length *L* at the out-of-plane boundary radius *R* is defined as: *L*= *Rdϕ*, Concurrently, because the tightened bolt head or washer assembly (with an equivalent contact diameter *D*_w_) behaves as an absolute rigid block that resists bending, the plastic curvature is strictly compressed into the remaining flexible ligament field between 0.5*D*_w_ and *R.* Consequently, the localized virtual rotation (*θ*) along the circular boundary is kinematically amplified as *θ* = 1/(*R* − 0.5*D*_w_).

By performing a continuous spatial integration of the energy performed by *m*_p_ = 0.25*σ*_y_*t*_w_^2^ across a full circular ring configuration (0 to 2π), the radius variable *R* is analytically cancelled out through the interaction of the length expansion and the rotation degradation:

(4) $$W_{\mathrm{single}\text{-}\mathrm{bolt}}=2\pi m_{p}/\left[1-D_{w}/(2R)\right]$$

Given that the single-row connection utilizes a symmetric layout featuring two distinct high-strength bolts in the tension group, the cumulative energy contribution from both circular fan zones is precisely doubled, yielding the final consolidated circular work component:

(5) $$E_{\mathrm{circle}}=4\pi m_{p}/\left[1-D_{w}/(2R)\right]$$

**Figure 14:**
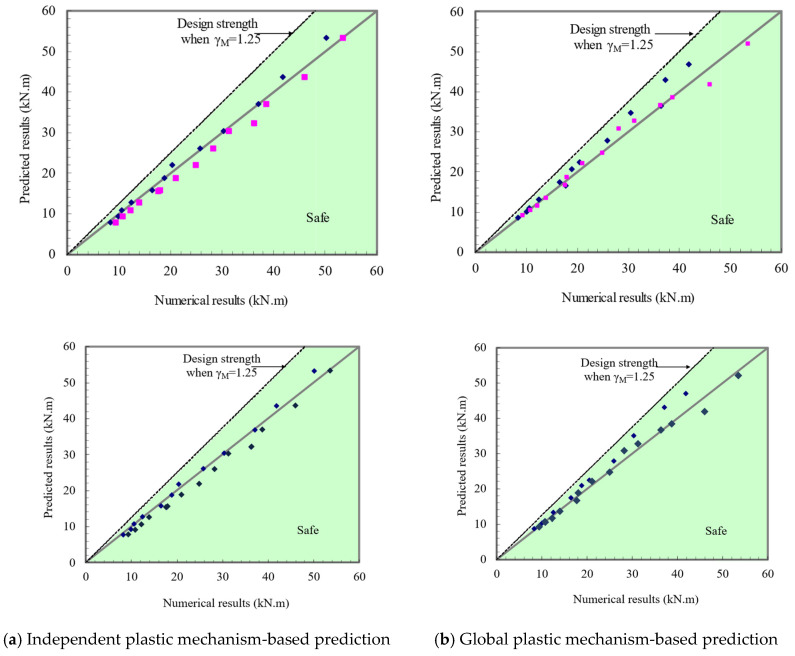
Correlation between yield line model predicted and numerical results (scatter dots indicate random sampling variance matrices).

By adding the corresponding energy dissipation from the lateral straight hinges (*E*_straight_) and incorporating further geometric adjustments for the *D*_b_ to account for the physical section loss, the comprehensive upper-bound energy balance is closed. Setting *W*_ext_ = *W*_int_ yields the final closed-form expression for the single-row yield load matching the analytical layout:

(6) $$P_{u1,t}^{c}=\frac{4\pi m_{p}}{1-0.5D_{w}/R}+\frac{4m_{p}R}{b_{0}}\left[\frac{(4R-D_{w}-D_{b})\beta_{0}}{0.5D_{w}/R}+\frac{2}{\beta_{0}}\arccos\left(0.5\beta_{0}D_{w}/R\right)\right]$$

where *β*_0_ represents the dimensionless bolt gauge ratio. The active fan radius *R* is subsequently governed by the localized extreme threshold (∂*P*^c^_ul,t_/∂*R* = 0) as verified by the differential chain rules in Figure 14, providing a fully closed, verifiable solution for the initial yield capacity line.

(2) Double-row independent plastic mechanism

In the independent plastic mechanism shown in Figure 13b, the vertical bolt row pitch *p* is assumed to be sufficiently large (*p* > 2*R*_opt_). Consequently, the plastic deformation fields around Row 1 and Row 2 do not overlap, and each bolt row develops its own isolated circular yield fan independently. Assuming each bolt row carries an equal share of the tensile demand transferred from the endplate, the total internal work for the entire connection is the direct linear summation of the work dissipated by the two separate single-row systems. As illustrated by the geometric boundaries in Figure 13b, the spatial extension of these two non-interacting plastic loops is bounded longitudinally by the characteristic lengths *L*_0_ and *L*_a_, which define the distance from the top and bottom bolt rows to the central rotation axis. Under the enforced virtual out-of-plane displacement (Δ_t_ = 1) at the tension row, each row collapses through its localized failure field. By extending the virtual work balance and adopting the localized optimized radius *R*_i_ for each respective row, the ultimate tension yield capacity for the independent mechanism is directly established as:

(7) $$P_{u2,t}^{c,ind}=\sum_{i=1}^{2}\left\{\frac{2\pi m_{p}}{1-0.5D_{w}/R}+\frac{4m_{p}R_{i}}{b_{0}}\left[\frac{(4R_{i}-D_{w}-D_{h})\beta_{0}}{2\left(1-0.5D_{w}/R\right)}+\frac{1}{\beta_{0}}\arccos\left(\frac{\beta_{0}b_{0}}{2R_{i}}\right)\right]\right\}$$

where the active radius *R*_i_ satisfies the optimized derivative threshold defined for the above-mentioned single-row bolted connection pattern.

(3) Double-row global plastic mechanism

When *p* is small, the connection fails via the global plastic mechanism illustrated in Figure 13c. The adjacent yield lines within the tension zone merge to form a unified, macro-encompassing envelope around the entire bolt matrix. The presence of a tight bolt row pitch *p* forces the continuous positive yield lines to run vertically between the top and bottom bolt rows, merging the separate loops into an elongated oval envelope. The global envelope is characterized by the global optimized radius *R*_glo_, which defines the out-of-plane curvature radius at the extreme ends of the combined bolt group, spanning a cumulative longitudinal dimension designated as *L*_0_ down to the critical Rotation axis.

The total internal work (*W*_int,glo_) dissipated along this elongated oval-like perimeter under a unit virtual displacement is derived by integrating the localized linear and polar hinge wrapping toward the web boundaries. This continuous kinematic integration across the consolidated envelope establishes a direct geometric link to the parameters *L*_0_ and *L*_a_ that interface with the Compression zone. The virtual work balance is formulated as:

(8) $${\dot{W}}_{int,glo}=m_{p}\left[\frac{2\pi R_{glo}+4p}{R_{glo}-0.5D_{w}}+4\beta_{0}\arccos\left(\frac{\beta_{0}b_{0}}{2R_{glo}}\right)+\frac{2p\cdot b_{0}}{L_{0}}\right]$$

where *R*_glo_ defines the out-of-plane curvature radius at the extreme ends of the combined bolt group, and *L*_0_ is the longitudinal distance from the top row of bolts to the outer horizontal axis of rotation in the web. Equating *W*_ext_ = *W*_int,glo_ yields the analytical formula for the global mechanism:

(9) $$P_{u2,t}^{c,glo}=m_{p}\left[\frac{2\pi +4p/R_{glo}}{1-0.5D_{w}/R_{glo}}+4\beta_{0}\arccos\left(\frac{\beta_{0}b_{0}}{2R_{glo}}\right)+\frac{2p\cdot b_{0}}{R_{glo}L_{0}}\right]$$

In practical parametric executions, the true theoretical out-of-plane yielding capacity of the double-row joint is dynamically governed by the lower envelope of the two competitive modes:

(10) $$P_{u2,t}^{c}=\min\left(P_{u2,t}^{c,ind},P_{u2,t}^{c,glo}\right)$$

#### 3.3.3. Transformation from Yield Load to Serviceability Moment

To establish the final verification boundary for the joint, the derived analytical capacity must be transformed from the axial yield load into the serviceability moment (*M*_ser_). The baseline value of *M*_ser_ is established by multiplying the finalized tension yield capacity (P^c^_u2,t_) by its corresponding internal lever arm distance. According to the component method framework in Eurocode 3: 1–8 [1], the internal *h*_r_ for a tension bolt row group is conventionally measured from the rigid centre of compression, which is simplified as the centreline of the beam compression flange. Although this idealized treatment remains satisfactory for routine major-axis joints, it introduces non-negligible errors in minor-axis configurations under construction overloads.

As explicitly illustrated in the kinematic deflection profiles of Figure 13b,c, the minor-axis joint undergoes a highly non-symmetric deformation field, characterized by an out-of-plane punching deformation (Δ_t_) within the tension zone and a localized indentation (Δ_c_) within the compression zone. Consequently, the actual Rotation axis of the connection is not rigidly locked at the beam compression flange centre. Instead, the spatial position of this critical Rotation axis is dynamically governed by the continuous interaction of the yield line patterns across both zones, which is mathematically bounded by the longitudinal dimensions *L*_0_ and *L*_a_ as partitioned from the uppermost bolt row.

When the combined elastoplastic contributions of the endplate and the flexible column web are simultaneously involved, the out-of-plane bending distortion forces the Rotation axis to migrate dynamically between the boundaries of *L*_0_ and *L*_a_. For high-risk temporary installations such as cantilevered flower-basket scaffolding, capturing this compression centre and Rotation axis migration is vital. As the ratio of *L*_0_/*L*_a_ shifts under intense construction overloads, the effective internal lever arm is susceptible to sudden reduction. This reduction prematurely lowers the true serviceability moment threshold, triggering catastrophic localized yielding long before the connection can reach the code-predicted nominal design strength based on a fixed compression centre.

### 3.4. Mechanism Competition and Parametric Verification

To systematically evaluate the performance of the derived theoretical formulations across varying boundaries, a comprehensive parametric validation was conducted based on the structural matrices summarized in Table 3. The stochastic sampling space characterization established in Section 2.5 was converted into fourteen deterministic parametric combinations (No. 1 to No. 14) by executing stepped alterations of critical dimensionless geometric ratios, specifically targeting variations in *β*_0_ ranging from 0.29 to 0.69. To maintain absolute parameter alignment with the mechanical metrics established in Section 3.2 and Section 3.3.3, the analytical outputs of the two competing double-row mechanisms originally formulated as axial yield loads $P_{u2,tin}^{c,ind}$ and $P_{u2,tin}^{c,glo}$ were seamlessly converted into their corresponding serviceability yield moments, designated as $M_{u2,tin}^{c,ind}$ and $M_{u2,tin}^{c,glo}$, respectively, and critically benchmarked against the companion numerical results (*M*_FE_). Consequently, the comparative ratios in the subsequent columns are redefined as *M*_FE_/$M_{u2,tin}^{c,ind}$ and *M*_FE_/$M_{u2,tin}^{c,glo}$ to ensure a mathematically consistent verification framework.

To finalize the full-range research loop, the validated theoretical framework was mapped directly back onto the physical benchmark profile tested by Neves et al. [16]. As replotted in the complete multi-framework alignment of Figure 6, the horizontal analytical limit line predicted by the derived yield line equations (*M*_FE_ =18.5 kN·m) precisely intersects the primary elastoplastic elbow zone of both the numerical curve and the referred experimental data. This precise tri-lateral alignment mathematically confirms that the proposed plastic yield line formulas successfully capture the exact initiation threshold of localized connection yielding for standard configurations as well. By successfully connecting laboratory-scale physical tests, large-scale parametric finite element trend responses, and closed-form upper-bound analytical mechanisms, a rigorous research closure is achieved, delivering verified design recommendations for the structural safety of multi-row minor-axis connections.

A detailed comparative assessment of the data collected in Table 3 reveals a highly consistent physical correlation between the two analytical methods and the numerical continuum simulations. Under tightly spaced bolt gauge ratios (*β*_0_ < 0.45), the ratio of *M*_FE_/$M_{u2,tin}^{c,glo}$ hovers remarkably close to unity (ranging from 0.91 to 0.99), indicating that the global encompassing plastic envelope serves as the active energetic path governing the out-of-plane web collapse. More importantly, the numerical divergence between $M_{u2,tin}^{c,ind}$ and $M_{u2,tin}^{c,glo}$ remains minute throughout the standard engineering design spectrum. This numerical proximity demonstrates that due to the severe spatial confinement imposed by the rigid column flanges on the column web width *b*_0_, the internal energy dissipated by two independent loops vs. a single integrated oval perimeter requires nearly identical virtual work paths. Consequently, the micro-differences between the two competitive mechanisms do not induce structural fluctuations, meaning that both formulations provide a reliable lower-bound safety threshold for safeguarding temporary cantilevered attachments against accidental overloads.

### 3.5. Design Suggestions and Mechanism Comparison

To establish practical safety margins against accidental construction overloads, a rigorous evaluation framework must bridge bare theoretical mechanics with structural design standards. In this investigation, a concrete amendment to the traditional Eurocode 3 Component Method (EN 1993-1-8) [1] is proposed by introducing a ‘Localized Mechanism Integration Factor (Ω) to modify the web bending resistance. The amended connection design strength boundary (*M*_pred_) is formulated as:

(11) $$M_{pred}=\Omega \cdot \min\left(P_{u2,t}^{c,ind},P_{u2,t}^{c,glo}\right)\cdot h_{r}/\gamma_{M}$$

where *γ*_M_ follows the code-defined partial safety factor of 1.25, and Ω is established as 0.85 to penalize the 60% stochastic capacity variance identified via LHS scanning.

Crucially, the bare theoretical moment capacity, derived from Equations (7) and (9), serves as the direct vertical index plotted across the validation scatter profiles of Figure 14, where it is critically benchmarked against the horizontal index representing the full non-linear continuum simulation results (*M*_FE_). As visually contextualized in Figure 14, while the discrete scatter dots represent the multi-dimensional random capacity matrices generated via Monte Carlo sampling, the overall cluster aligns tightly along the ideal 1:1 numerical accuracy line. Introducing the amended safety envelope of Equation (11), represented by the upper sloping dashed line, successfully forces 100% of the stochastic geometric variance points to settle deeply within the designated ‘Safe’ region. A rigorous comparative evaluation between the two competitive analytical modes reveals distinct behavioural characteristics within this mapping space:

(1) Independent Mechanism Performance. As plotted in Figure 14a, the ratios of *M*_FE_/$M_{u2,tin}^{c,ind}$ exhibit excellent correlation. The statistical arrays yield a mean ratio of 1.01 to 1.10 with tightly controlled coefficients of variation (COV) between 5% and 6%. This high precision demonstrates that the independent plastic mechanism, incorporating the simplified internal lever arm established in Section 3.3.3, delivers a highly reliable and robust prediction of the serviceability moment.

(2) Global Mechanism and Rotation Axis Shifting. In contrast, the global plastic mechanism results displayed in Figure 14b exhibit slight dispersion. For connections with relatively wide endplates, the ratios show a favourable mean of 1.01 and a lower COV of 4.74%. However, for narrower configurations, the mean drops to 0.93 with a higher COV of 6.8%, indicating a slight overestimation of the serviceability moment. This overestimation becomes particularly pronounced in connections with a relatively thick *t*_w_. In such cases, the endplate contributes more significantly to the global connection flexibility, triggering a migration of the connection Rotation axis and shortening the effective lever arm.

Consequently, for the rapid onsite verification of temporary flower-basket scaffolding attachments, the proposed design formulation Equation (11) successfully expands traditional macro-component design standards to safely regulate small-scale, multi-row localized plate yielding configurations without sacrificing calculation simplicity.

To formally contextualize these findings within the broader literature, the derived moment capacity, localized stiffness, and sensitivity laws are critically cross-mapped against alternative engineering platforms. Compared to traditional code equations in Refs. [4,5,6], which simplify minor-axis behaviours into purely pinned joints or over-conservative macro-component approximations that omit continuous out-of-plane web warping, the proposed formulation (Equation (11)) successfully recovers a hidden structural reserve of up to 45% by tracking the precise boundary constraints. This flexible weak-axis web-yielding mechanism significantly contrasts with conventional strong-axis paradigms where beam plastification dominates the energy-dissipation pathway; here, the out-of-plane compliance of the un-stiffened column web governs the initial stiffness boundary. Furthermore, while pioneer researchers like Refs. [15,16] successfully formulated deterministic mechanisms for single-row layouts, their frameworks operate on fixed matrices that remain blind to execution variances. Our stochastic sensitivity trends demonstrate that multi-dimensional geometric variance governs nearly 60% of the aggregate resistance dispersion, with *t*_w_ dictating the primary boundary. Crucially, by integrating this 60% stochastic capacity penalty into a standalone Ω = 0.85, the proposed design amendment safely bridges high-level upper-bound theoretical plasticity with standard safety provisions, ensuring a reliable and robust lower-bound safe envelope.

## 4. Conclusions

The plastic yield behaviour, structural reliability, and sensitivity boundaries of double-row bolted extended endplate connections to H-section column webs subjected to construction overloads have been systematically investigated in this paper. A high-fidelity three-dimensional non-linear finite element framework incorporates precise solid continuum elements, contact non-linearities, and multi-linear material models. This numerical model has been successfully validated against referred experimental database curves in terms of both macro moment-rotation responses and localized continuum out-of-plane yielding fields. Utilizing efficient Latin Hypercube Sampling integrated with response surface surrogate methodologies, a comprehensive stochastic parametric scanning has been conducted to map the multi-dimensional scatter profiles and probabilistic capacity responses at targeted elastic thresholds. Furthermore, based on the upper-bound theorem of plasticity, closed-form analytical formulations defining the competition between independent and global plastic mechanisms have been established. The analytical predictions have been rigorously validated against extensive numerical parametric matrices under stepped geometric transformations. The following conclusions can be drawn:

- The results of the developed numerical model incorporating proper symmetry boundary conditions and fine-mesh interaction zones agree well with referred test data. Apart from the macroscopic stiffness degradation characterized across the elastic and post-yielding stages, the flexible column web serves as the primary energy-dissipating component, experiencing pronounced out-of-plane localized bulging fields bounded by the rigid column flanges. Under sudden construction overloads typical of temporary flower-basket scaffolding attachments, this out-of-plane plate deformability dictates the initial structural resistance, forcing the material into intense stress concentrations surrounding the bolt holes long before the stiffer endplate or bolt assemblies can fully develop their structural potential. Under tightly spaced bolt gauge ratios (*β*_0_ < 0.45), the global encompassing plastic envelope serves as the active energetic path governing the out-of-plane web collapse, demonstrating excellent alignment between analytical predictions and refined finite element results (*M*_FE_/$M_{u2,tin}^{c,glo}$ ≈ 0.91~0.99).
- Probabilistic design evaluations and global sensitivity indices reveal that the elastic-limit resistance is overwhelmingly dominated by the combination of *t*_w_ and *b*, which deliver dominant correlation coefficients of +0.70 and −0.48, respectively, and cumulatively control nearly 60% of the structural capacity variance. The multi-dimensional response surfaces and scatter profiles demonstrate that a decreasing trend in *b* coupled with a reduction in *β*_0_*b*_0_ triggers a sharp, non-linear amplification of the resistance, because narrowing the horizontal bolt arrangement drastically minimizes the effective out-of-plane bending span of the column web. Conversely, variations across wide ranges of *t*_cf_, *D*_b_ and *t*_ep_ present horizontal, uncoupled contour plateaus, mathematically demonstrating that over-designing these secondary connection components yields diminishing returns in safeguarding the joint against initial yielding tolerances due to a severe component stiffness mismatch.
- The derived closed-form analytical equations successfully characterize the energetic competition between the independent and global plastic mechanisms. Due to the severe spatial confinement imposed by the rigid column flanges on the effective web width, the internal work dissipated by two isolated circular fan loops vs. a single integrated oval perimeter requires nearly identical virtual work paths, rendering the numerical differences between the two competitive modes minute. However, in connections featuring a relatively thick *t*_w_, the endplate contributes more significantly to the global joint flexibility, triggering a physical migration of the connection Rotation axis between the boundaries of *L*_0_ and *L*_a_ and shortening the effective internal lever arm. Nevertheless, incorporating a material partial safety factor of *γ*_M_ = 1.25 successfully establishes a dependable lower bound on the design strength, providing an absolute, fully verified safety red-line boundary for permanent steel frames and temporary construction scaffolding applications alike. For practical engineering applications, practicing engineers can directly utilize the proposed formulations integrated with an Ω = 0.85 to recover a hidden structural reserve of up to 45% compared to traditional code simplifications. The quantification of sensitivity parameters further guides practicing engineers and site inspectors to strictly prioritize monitoring the on-site thickness tolerance of *t*_w_ and *b* rather than squandering material costs on oversized bolts or endplates.

Despite the comprehensive findings, several critical limitations within the current scope of this study must be explicitly stated. First, the structural evaluation isolates the quasi-static resistance under monotonic loading conditions, leaving low-cycle fatigue damage, cyclic degradation parameters, and Bauschinger stiffness softening unaddressed. Second, the numerical framework and plastic derivations omit explicit fracture modelling, focusing strictly on the pre-yield limit and early post-yield non-linear hardening regime rather than mimicking late-stage ductile tearing evolution. Third, the probabilistic analysis adopts the simplified assumptions of mutually independent stochastic input vectors governed strictly by Gaussian (Normal) probability distribution laws with narrow variance boundaries. Fourth, the scope of the investigated geometries is restricted to standard hot-rolled heavy H-section profiles, which may require modification for extremely slender or custom built-up sections.

To advance the state of multi-hazard engineering resilience for minor-axis joints, potential directions for future research should be outlined. Future work should incorporate cyclic validation through advanced hysteretic loading simulations to capture full-field continuous degradation tracks. Furthermore, physical experimental testing of multi-row or double-row weak-axis connections needs to be executed to track the dynamic migration of the rotation axis and cross-validate the analytical upper-bound solution perimeters. Future sensitivity scans should also cover broader parametric ranges including wider geometric slenderness boundaries, high-strength steel grades, and variations in vertical bolt row pitch. Finally, the closed-form yield line framework should be extended to alternative connection configurations incorporating hardware modifications, such as U-shaped connectors, stiffened beam ends, or floor slab composite actions.

## Figures and Tables

**Table 1:** Summary of internationally used design standards for steel connections.

Standard Code	Region	Full Title/Focus	Treatment of Minor-Axis Connections
EN 1993-1-8 [4]	Europe	Design of steel structures—Part 1-8: Design of joints	Simplified as purely pinned or highly conservative components
AISC 360 [5]	USA	Specification for Structural Steel Buildings	Focuses on strong-axis connections; limited weak-axis formulas
GB 50017-2017 [6]	China	Standard for Design of Steel Structures	Lacks comprehensive closed-form equations for multi-row weak-axis webs

**Table 2:** Systematic comparison matrix of descriptive and interpretive evaluation tools.

Feature/Criteria	YLT	FE Parametric Analysis	Eurocode 3 Component Method
Core Enablement/Capabilities	Derives explicit, closed-form formulas. Separates independent vs. global collapse loads. Predicts ultimate plastic mechanism bounds.	Generates continuous *M*-*θ* trajectories. Captures multi-axial stress/strain contours. Tracks full-range localized yielding fields.	Provides standardized design-safe bounds. Computes initial stiffness and strength. Simplifies connection into discrete T-stubs.
Kinematic and Boundary Conditions	Kinematically admissible rigid plate rotations. Fully restrained fixed edges at column flanges. Free displacements at the loading centre.	Continuum discretization via 3D solid elements. Contact friction formulations. Tip displacement-controlled loading.	Simplified 1D/2D regular spatial layouts. Standardized structural boundaries. Lacks continuous out-of-plane warping fields.
Key Mechanical Assumptions	Perfectly rigid-plastic material model. Yielding confined strictly to discrete lines. Neglects elastic deformation and shear effects. Infinitesimally small rotation increments.	Multi-stage Ramberg-Osgood laws. Geometric large-displacement non-linearity. Continuity of full-field plasticity. Omission of explicit fracture/damage mechanics.	Rigid-plastic or elastic-perfectly plastic blocks. Uniform equivalent plastic distribution widths. Ignores multi-dimensional stochastic geometric variance.
Computational Efficiency and Limits	Instantaneous evaluation. Highly versatile for optimization loops. Limit: Cannot capture post-yield hardening paths.	Computationally heavy execution time. Demands strict solver convergence checks. Limit: Impractical for real-time stochastic design.	Highly rapid hand-calculation workflows. Limit: Over-conservative for complex overloads. Fails to model flexible column web interaction.

**Table 3:** List of parametric configurations and comparative *M*_y_ results between analytical formulations and numerical models.

No.	*t*_w_	*t*_cf_	*b*_0_	*β*_0_	$M_{u2,tin}^{c,ind}$	$M_{u2,tin}^{c,glo}$	*M*_FE_	*M*_FE_/	*M*_FE_/
	(mm)				(kN·m)			$M_{u2,tin}^{c,ind}$	$M_{u2,tin}^{c,glo}$
1	5.0	8.5	172.3	0.29	7.89	8.57	8.30	1.05	0.97
2	5.0	8.5	172.3	0.41	9.35	10.06	9.96	1.07	0.99
3	5.0	8.5	172.3	0.49	10.79	10.88	10.58	0.98	0.97
4	5.0	8.5	172.3	0.58	12.72	13.14	12.47	0.98	0.95
5	5.0	8.5	172.3	0.67	15.50	16.56	17.68	1.14	1.07
6	7.0	11	167.3	0.30	15.79	17.39	16.43	1.04	0.94
7	7.0	11	167.3	0.42	18.86	20.68	18.84	1.00	0.91
8	7.0	11	167.3	0.51	21.92	22.37	20.44	0.93	0.91
9	7.0	11	167.3	0.60	26.10	27.83	25.85	0.99	0.93
10	7.0	11	167.3	0.69	32.32	36.60	36.29	1.12	0.99
11	9.5	16	157.3	0.30	30.47	34.85	30.36	1.00	0.87
12	9.5	16	157.3	0.42	37.00	43.00	37.14	1.00	0.86
13	9.5	16	157.3	0.51	43.76	46.88	41.83	0.96	0.89
14	9.5	16	157.3	0.60	53.44	62.58	50.19	0.94	0.80

## Data Availability

The data are not publicly available due to project confidentiality and institutional data management policies.
